# Antileishmanial Effects of Acetylene Acetogenins from Seeds of *Porcelia macrocarpa* (Warm.) R.E. Fries (Annonaceae) and Semisynthetic Derivatives

**DOI:** 10.3390/molecules27030893

**Published:** 2022-01-28

**Authors:** Ivanildo A. Brito, Fernanda Thevenard, Thais A. Costa-Silva, Samuel S. Oliveira, Rodrigo L. O. R. Cunha, Emerson A. de Oliveira, Patricia Sartorelli, Rafael C. Guadagnin, Maiara M. Romanelli, Andre G. Tempone, João Henrique G. Lago

**Affiliations:** 1 Human and Natural Science Center, Universidade Federal do ABC, São Paulo 09210-580, Brazil; ivanildo.brito@ufabc.edu.br (I.A.B.); fernandabr797@hotmail.com (F.T.); tha_isbio@yahoo.com.br (T.A.C.-S.); quimiossoliveira@gmail.com (S.S.O.); rodrigo.cunha@ufabc.edu.br (R.L.O.R.C.); 2 Departament of Chemistry, Universidade Federal de São Paulo, São Paulo 09972-270, Brazil; dupontemerson@hotmail.com (E.A.d.O.); psartorelli@unifesp.br (P.S.); rcguadagnin@gmail.com (R.C.G.); 3 Department of Parasitology, Instituto Adolfo Lutz, São Paulo 01246-902, Brazil; ma.romaneli@gmail.com (M.M.R.); andre.tempone@ial.sp.gov.br (A.G.T.)

**Keywords:** *Porcelia macrocarpa*, acetylene acetogenins, *Leishmania (L.) infantum*, leishmaniasis

## Abstract

As part of our continuous studies involving the prospection of natural products from Brazilian flora aiming at the discovery of prototypes for the development of new antiparasitic drugs, the present study describes the isolation of two natural acetylene acetogenins, (*2S*,*3R*,*4R*)-3-hydroxy-4-methyl-2-(*n*-eicos-11′-yn-19′-enyl)butanolide (**1**) and (*2S*,*3R*,*4R*)-3-hydroxy-4-methyl-2-(*n*-eicos-11′-ynyl)butanolide (**2**), from the seeds of *Porcelia macrocarpa* (Warm.) R.E. Fries (Annonaceae). Using an ex-vivo assay, compound **1** showed an IC_50_ value of 29.9 μM against the intracellular amastigote forms of *Leishmania (L.) infantum*, whereas compound **2** was inactive. These results suggested that the terminal double bond plays an important role in the activity. This effect was also observed for the semisynthetic acetylated (**1a** and **2a**) and eliminated (**1b** and **2b**) derivatives, since only compounds containing a double bond at C-19 displayed activity, resulting in IC_50_ values of 43.3 μM (**1a**) and 23.1 μM (**1b**). In order to evaluate the effect of the triple bond in the antileishmanial potential, the mixture of compounds **1** + **2** was subjected to catalytic hydrogenation to afford a compound **3** containing a saturated side chain. The antiparasitic assays performed with compound **3**, acetylated (**3a**), and eliminated (**3b**) derivatives confirmed the lack of activity. Furthermore, an in-silico study using the SwissADME online platform was performed to bioactive compounds **1**, **1a**, and **1b** in order to investigate their physicochemical parameters, pharmacokinetics, and drug-likeness. Despite the reduced effect against amastigote forms of the parasite to the purified compounds, different mixtures of compounds **1 + 2**, **1a + 2a**, and **1b + 2b** were prepared and exhibited IC_50_ values ranging from 7.9 to 38.4 μM, with no toxicity for NCTC mammalian cells (CC_50_ > 200 μM). Selectivity indexes to these mixtures ranged from >5.2 to >25.3. The obtained results indicate that seeds of *Porcelia macrocarpa* are a promising source of interesting prototypes for further modifications aiming at the discovery of new antileishmanial drugs.

## 1. Introduction

Leishmaniasis is a neglected tropical disease caused by the protozoan parasite *Leishmania* sp. Different forms of this disease can be found, and the visceral is responsible for the high lethality of this disease [1]. The treatment of leishmaniasis is limited and involves using toxic drugs such as antimonial derivatives, amphotericin B, and miltefosine. Based on this scenario, the search for new hit compounds is crucial, and natural products can provide inspiring molecules for drug discovery studies against this neglected tropical diseases [2,3].

**Figure 1:**
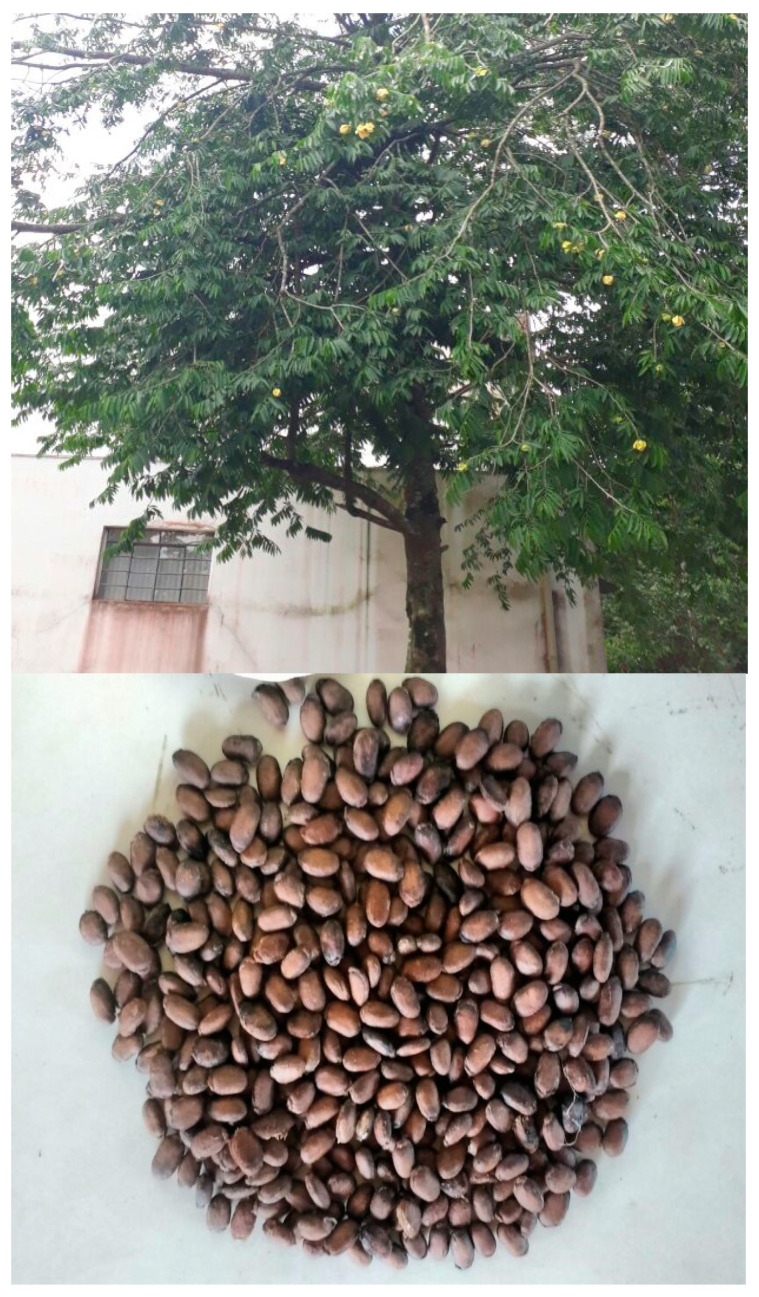
Adult tree and dried seeds obtained from fresh fruits of *P. macrocarpa*.

*Porcelia macrocarpa* (Warm.) R. E. Fries (Annonaceae—Figure 1) is a tropical plant with restricted occurrence in the Atlantic Forest and “Cerrado” (savanna like) regions of Brazil. Fruits of this plant have been consumed *in natura* due to their sweet flavor, but the seeds are frequently discarded [4]. 

Aiming to discover new natural products with antiprotozoal activity, different studies with *P. macrocarpa* have previously been performed. Initially, the presence of anti-trypanosomal fatty acid containing a diyn system was reported, but this compound exhibited activity only against trypomastigote forms of *Trypanosoma cruzi* [5]. In a second study, chemically related fatty acids containing enyn and diyn systems were isolated from flowers and exhibited anti-*T. cruzi* activity against trypomastigotes [6]. More recently, new acetylene acetogenins were obtained from seeds of *P. macrocarpa* with anti-*T. cruzi* activity, especially against amastigote forms [7]. Additionally, these compounds induced alterations in the plasma membrane permeability and in the electric potential of the mitochondrial membrane as well as in the reactive oxygen species (ROS) levels of the parasite [6,7]. Despite the effect against *T. cruzi*, there is one unique study evaluating the effect of different acetogenins and fatty acid from *P. macrocarpa* against intracellular forms of *Leishmania (L.) infantum*, which is the most relevant form of the parasite [8]. 

In continuation to our studies, in the present work, two acetylene acetogenins were isolated from seeds of *P. macrocarpa* and evaluated against amastigote forms of *L. (L.) infantum*. Additionally, these compounds were subjected to different modifications, including acetylation, and conversions to elimination and hydrogenation reactions products, to discover important check critical pharmacophoric groups in these chemically related compounds. Furthermore, considering that natural acetogenins were obtained in a mixture and showed higher potential against *L. (L.) infantum* than purified compounds, we also evaluated them as a mixture of natural products or as semisynthetic derivatives.

## 2. Results and Discussion

*Porcelia macrocarpa* is a Brazilian plant that produces different bioactive metabolites such as terpenoids, alkaloids, acetylene fatty acids, and acetogenins [5,6,7,8,9,10,11]. In this work, two acetylene acetogenins were isolated from seeds of this plant using several chromatographic techniques, and their structures were confirmed as (*2S*,*3R*,*4R*)-3-hydroxy-4-methyl-2-(*n*-eicos-11′-yn-19′-enyl)butanolide (**1**) and (*2S*,*3R*,*4R*)-3-hydroxy-4-methyl-2-(*n*-eicos-11′-ynyl)butanolide (**2**) by NMR and ESI-HRMS spectral analysis (see Supplementary Material) and comparison with data described in the literature [11]. 

**Figure 2:**
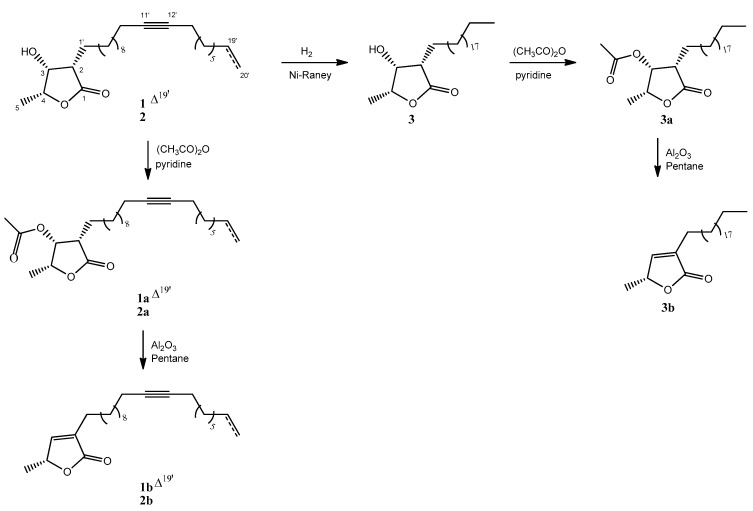
Chemical structures of natural acetogenins (**1** and **2**) and semisynthetic derivatives (**1a**, **1b**, **2a**, **2b**, **3, 3a**, and **3b**).

Aiming to establish relationships between the chemical structures and the antiparasitic activity, these compounds were subjected to a sequence of reactions including hydrogenation, acetylation, and elimination to afford compounds (*2S*,*3R*,*4R*)-3-acetoxy-4-methyl-2-(*n*-eicos-11′-yn-19′-enyl)butanolide (**1a**), *4R*-methyl-2-(*n*-eicos-11′-ynyl)but-2-enolide (**1b**), (*2S*,*3R*,*4R*)-3-hydroxy-4-methyl-2-(*n*-eicos-11′-ynyl)butanolide (**2a**), *4R*-methyl-2-(n-eicosyl)but-2-enolide (**2b**), (*2S*,*3R*,*4R*)-3-hydroxy-4-methyl-2-(*n*-eicosyl)butanolide (**3**), (*2S*,*3R*,*4R*)-3-acetoxy-4-methyl-2-(*n*-eicosyl)butanolide (**3a**), and *4R*-methyl-2-(*n*-eicosyl)but-2-enolide (**3b**), as shown in Figure 2. Structures of these compounds were also established by analysis of NMR and HRESI-MS spectral data (see Supplementary Material).

As previously reported [5,6,7], *P. macrocarpa* is an important source of natural antiprotozoal natural products including acetylenic fatty acids and acetogenins with anti-*T. cruzi* potential. Recently, our group reported the occurrence of different acetylene acetogenins and fatty acids with anti-*Leishmania* activity, especially against the intracellular amastigote forms [8]. 

As part of this continuous study, the anti-*L. (L.) infantum* activity of two natural compounds (**1** and **2**) was performed against amastigotes forms of this parasite, whereas the toxicity of these compounds was determined using NCTC cells. The obtained results (Table 1) demonstrated that compounds **1** and **2** induced no mammalian cytotoxicity against NCTC cells to the highest tested concentration (200 μM). When tested against *L. (L.) infantum* amastigotes, compound **1** displayed activity with an IC_50_ value of 29.9 μM, while compound **2** was inactive (IC_50_ > 100 μM). This effect might be ascribed to the terminal double bond at C-19, which may play an important role in the activity. A similar effect was also observed for the hydrogenated derivative **3**, which showed no activity (IC_50_ > 100 μM). Considering the structure of γ-lactone unit and based on our previous studies describing the higher activity of conjugated natural acetogenins [7], the preparation of an α,β-unsaturated system was planned via acetylation of the hydroxyl group at C-3 followed by the elimination of acetic acid over alumina chromatographic column. 

This approach afforded acetyl (**1a** and **2a**) and eliminated (**1b** and **2b**) derivatives from acetogenins **1** and **2** as well as from hydrogenated products (**3a** and **3b**). As a result, all derivatives prepared from compound **2** displayed no activity against amastigote forms of *L.(L.) infantum*. Considering the activity of acetogenin **1,** with an IC_50_ value of 29.9 μM, the acetylated compound **1a** exhibited lower activity (IC_50_ of 43.3 μM), while the eliminated product **1b** showed a similar potential of natural product (IC_50_ of 23.1 μM).

Finally, considering that compounds **1** and **2** were naturally obtained in a mixture of 2:1 and exhibited potent activity against amastigotes (IC_50_ of 8.4 μM), reduced toxicity (CC_50_ > 200 μM), SI > 23.8, and a superior efficacy when compared to the standard drug miltefosine (IC_50_ of 17.8 μM and CC_50_ of 116 μM), other different mixtures of compounds **1** and **2** were prepared. As observed in Table 1, mixtures composed of 1:1 and 1:2, with IC_50_ values of 13.6 and 19.4 μM, respectively, displayed lower potency than that at a 2:1 ratio. A similar effect was observed for the mixtures prepared with acetyl derivatives **1a** and **2a**, which showed an IC_50_ value for the mixture 2:1 of 12 μM and a SI > 16.7. An interesting effect was observed for the eliminated derivatives **1b** and **2b**, since mixtures at 2:1, 1:1, and 1:2 displayed IC_50_ values of 7.9, 10.5, and 18.2 μM, respectively, indicating the superior potential of that determined for standard drug miltefosine (IC_50_ of 17.8 μM).

**Figure 3:**
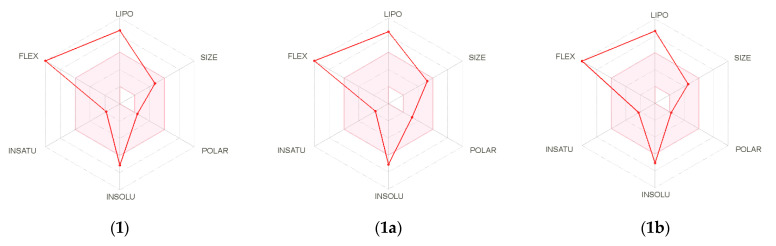
Bioavailability radar for bioactive compounds (**1)**, (**1a**), and (**1b**). The pink zone indicates the physicochemical space for oral bioavailability, and the red line indicates the oral bioavailability properties.

To evaluate the potential of bioactive compounds **1**, **1a**, and **1b** as new prototypes for the development of drugs against *L. (L.) infantum*, an *in-silico* analysis using the SwissADME online platform was performed. Using this approach, it was possible to investigate different properties of the tested compounds such as physicochemical parameters, pharmacokinetics, and drug-likeness. As an initial evaluation, the bioavailability radar (Figure 3) of tested compounds demonstrated similar characteristics with adherence to some parameters such as unsaturation, polarity, and size. However, the presence of the C_20_ side chain confers, to tested compounds, high lipophilicity and reduced solubilization in aqueous medium, as indicated by red lines outside of the physicochemical space in the bioavailability radar.

Additionally, other predicted proprieties such as pharmacokinetics and drug-likeness proprieties for compounds **1**, **1a**, and **1b** were calculated using the online platform SwissADME and are described in Table 2.

Based on the obtained data, bioactive compounds **1**, **1a**, and **1b** exhibited similar results to all evaluated parameters, especially physicochemical parameters (fraction Csp^3^, number of rotatable bonds, H-bond acceptors, and H-bond donors), lipophilicity (log *p*), water solubility, and GI absorption. Furthermore, tested compounds showed partial adherence to Lipinski’s rules-of-five (RO5), presenting one unique violation (log *p* > 4.15). The analysis of the binding to cytochrome-related isoenzymes CYP 450 indicated that all tested compounds could be considered not promiscuous molecules since they do not cause any inhibition of CYP2C1, CYP2D6, and CYP3A4 but only to CYP1A2 and CYP2CA. Finally, no alerts were evidenced for PAINS to tested compounds.

Therefore, the obtained results suggested that the double bond and C-19 are crucial structural features associated with antileishmanial activity. However, the effect against the parasite is intensified when mixtures of natural compounds **1** and **2** are tested. As detected, the natural mixture of these compounds at 2:1 showed to be the best proportion of natural products, suggesting that the original mixture of acetogenins obtained from *P. macrocarpa*, easily obtained after simple chromatographic steps, could be considered an important source of new prototypes to the development of new derivatives to be tested against amastigotes of *L. (L.) infantum*.

## 3. Materials and Methods 

### 3.1. General Experimental Procedures

Column chromatographic procedures were performed using silica gel 60 (Merck—Darmstadt, Germany) or Sephadex LH-20 (Sigma-Aldrich—St. Louis, MO, USA), whereas analytical TLC separations were conducted using silica gel F_254_ (Merck—Darmstadt, Germany). HPLC analysis was performed using a Dionex Ultimate 3000 chromatograph with a UVD-DAD-170 V as the detector, using a Luna Phenomenex C18 column (particle and pore size of 5 μm and 120 Å)—10 × 250 mm to semipreparative and 4.6 × 250 mm to analytical modes. Analytical grade solvents and reagents were used for every chromatographic procedure (Labsynth Ltd.a, SP, Brazil). NMR spectra were recorded on a Varian INOVA 500 (Palo Alto, CA, USA) operating at 500 and 125 MHz for ^1^H and ^13^C nuclei, respectively. Spectra were recorded using CDCl_3_ (Aldrich, St. Louis, MO, USA) as solvent and TMS as internal standard. ESI-HRMS spectra were recorded on a MicroTOF QII Bruker Daltonics (Billerica, MA, USA) spectrometer using positive or negative ionization modes. 

### 3.2. Plant Material 

*P. macrocarpa* fruits were collected in the Instituto de Botanica de São Paulo, São Paulo State, Brazil in June 2017 under coordinates—23°38′33.8″ S and 46°37′17.5″ W. The plant material was identified by Prof. Dr. Maria Claudia M. Young. A voucher specimen (SP76791) has been deposited in the Herbarium of Instituto de Botanica de São Paulo, SP, Brazil.

### 3.3. Extraction and Isolation

*P. macrocarpa* fruits were dried over 30 °C under 24 h, and the seeds were manually extracted. After this procedure, seeds were dried over 45 °C during 72 h, powdered, and the obtained material (600 g) was successively extracted using *n*-hexane (6 × 1000 mL) and CH_2_Cl_2_ (6 × 1000 mL) at room temperature. After evaporation of solvents at reduced pressure, 16 g of *n*-hexane and 32 g of CH_2_Cl_2_ extracts were obtained. Part of the CH_2_Cl_2_ extract (30 g) was applied to a silica gel column and eluted with a gradient mixture of EtOAc in *n*-hexane (9:1, 4:1, 7:3, 1:1, 3:7, and 1:9) to afford four groups (A to D) after TLC analysis. Fraction C (3013 mg) was subjected to column chromatography over silica gel eluted with a gradient mixture of EtOAc in *n*-hexane (4:1, 7:3, and 1:1) to afford eight groups (C1–C8) after TLC analysis. Fraction C4 (500 mg) was purified by HPLC (eluent ACN:H_2_O 9:1, flow rate 3.6 mL/min, detection at 220 nm) to afford pure compounds **1** (320 mg) and **2** (120 mg). 

*(2S*,*3R*,*4R)-3-Hydroxy-4-methyl-2-(*n*-eicos-11′-yn-19′-enil)butanolide* (**1**). Amorphous white solid, 100% purity by HPLC. ^1^H NMR (500 MHz, CDCl_3_), δ 5.79 (ddt, *J* = 16.9, 10.2 and 6.7 Hz, H-19′), 4.94 (m, H-20′), 4.43 (dq, *J* = 6.5 and 3.0 Hz, H-4), 4.24 (dd, *J* = 4.8 and 3.1 Hz, H-3), 2.55 (dt, *J* = 9.9 and 4.9 Hz, H-2), 2.11 (t, *J* = 6.8 Hz, H-10′ and H-13′), 2.02 (m, H-18′), 1.78 (m, H-1′), 1.63 (m, H-2′), 1.41 (d, *J* = 6.5 Hz, CH_3_-4), 1.26 (br s, H-3′ to H-9′ and H-14′ to H-17′). ^13^C NMR (125 MHz, CDCl_3_), δ 178.0 (C-1), 139.2 (C-19′), 114.2 (C-20′), 80.3 (C-12′), 80.2 (C-11′), 79.1 (C-4), 71.3 (C-3), 47.7 (C-2), 33.8 (C-18′), 28.7–29.6 (C-3′ to C-9′ and C-14′ to C-17′), 27.7 (C-2′), 23.4 (C-1′), 18.8 (C-10′ and C-13′), 13.8 (CH_3_-4). ESI-HRMS (positive mode) *m/z* 391.3227 [M + H]^+^ (calculated for C_25_H_43_O_3_ 391.3212). 

(2*S*,3*R*,4*R*)-3-Hydroxy-4-methyl-2-(*n*-eicos-11′-ynil)butanolide (**2**). Amorphous white solid, 100% purity by HPLC. ^1^H NMR (500 MHz, CDCl_3_) δ 4.43 (dq, *J* = 6.5 and 3.0 Hz, H-4), 4.24 (dd, *J* = 4.8 and 3.1 Hz, H-3), 2.55 (dt, *J* = 9.9 and 4.9 Hz, H-2), 2.11 (t, *J* = 6.8 Hz, H-10′ and H-13′), 1.78 (m, H-1′), 1.63 (m, H-2′), 1.41 (d, *J* = 6.5 Hz, CH_3_-4), 1.26 (br s, H-3′ to H-9′ and H-14′ to H-19′). ^13^C NMR (125 MHz, CDCl_3_), δ 178.0 (C-1), 139.2 (C-19′), 114.2 (C-20′), 80.3 (C-12′), 80.2 (C-11′), 79.1 (C-4), 71.3 (C-3), 47.7 (C-2), 33.8 (C-18′), 28.7–29.6 (C-3′ to C-9′ and C-14′ to C-17′), 27.7 (C-2′), 23.4 (C-1′), 18.8 (C-10′ and C-13′), 13.8 (CH_3_-4). ESI-HRMS (positive mode) *m/z* 393.3382 [M + H]^+^ (calculated for C_25_H_45_O_3_ 393.3369). 

### 3.4. Preparation of Semisynthetic Compounds

#### 3.4.1. Hydrogenation (Compound **3**)

In a high-pressure reactor (stainless steel) was added a mixture of compounds **1** + **2** (100 mg) dissolved in *n*-hexane (5 mL) and Ni-Raney catalyst (10 mg). After adding H_2_ (18 atm), the mixture was stirred for 3 h at 100 °C. Then, the catalyst was removed by filtration over a bed of celite. Purification of the product by silica gel chromatography (*n*-hexane/EtOAc 9:1) afforded compound **3** (80 mg, 78%).

(2*S*,3*R*,4*R*)-3-Hydroxy-4-methyl-2-(*n*-eicosanil)butanolide (**3**). Amorphous white solid, 100% purity by HPLC. ^1^H NMR (500 MHz, CDCl_3_) δ 4.44 (dq, *J* = 6.4 and 3.0 Hz, H-4), 4.31 (dd, *J* = 4.4 and 3.1 Hz, H-3), 2.57 (dt, *J* = 9.8 and 4.8 Hz, H-2), 1.82 (m, H-1′), 1.64 (m, H-2′), 1.43 (d, *J* = 6.5 Hz, CH_3_-4), 1.25 (br s, H-3′ to H-19′), 0.87 (t, *J* = 6.7 Hz, H-20′). ^13^C NMR (125 MHz, CDCl_3_) δ 177.6 (C-1), 79.9 (C-4), 71.4 (C-3), 47.7 (C-2), 32.0 (C-18′), 29.5–29.8 (C-3′ to C-17′), 27.7 (C-2′), 23.4 (C-1′), 22.8 (C-19′), 14.2 (C-20′), 13.8 (CH_3_-4). ESI-HRMS (positive mode) *m/z* 419.3480 [M + Na]^+^ (calculated for C_25_H_48_O_3_Na 419.3501).

#### 3.4.2. Acetylation (Compounds **1a**–**3a**)

Solutions of compounds **1** (15 mg), **2** (12 mg), or **3** (58 mg) were individually dissolved in pyridine (5 mL) and cooled to 0 °C. Acetic anhydride (5 mL) was added to each solution and was stirred for 15 h at room temperature. After adding cold H_2_O (20 mL), the residue was extracted with CHCl_3_ (3 × 15 mL). Organic layers were dried over Na_2_SO_4_, filtered, and concentrated. This procedure afforded **1a** (13 mg, 81%), **2a** (12 mg, 90%), and **3a** (51 mg, 80%).

(2*S*,3*R*,4*R*)-3-Acetoxy-4-methyl-2-(*n*-eicos-11′-yn-19′-enil)butanolide (**1a**). Amorphous white solid, 99% purity by HPLC. ^1^H NMR (500 MHz, CDCl_3_) δ 5.80 (ddt, *J* = 16.9, 10.2, and 6.6 Hz, H-19′), 5.59 (dd, *J* = 5.3 and 3.2 Hz, H-3), 4.96 (m, H-20′), 4.55 (dq, *J* = 6.4 and 3.2 Hz, H-4), 2.69 (dt, *J* = 9.6 and 5.2 Hz, H-2), 2.13 (t, *J* = 5.5 Hz, H-10′ and H-13′), 2.13 (s, CH_3_COO), 2.04 (m, H-18′), 1.81 (m, H-1′), 1.46 (m, H-2′), 1.31 (d, *J* = 6.5 Hz, CH_3_-4), 1.25 (br s, H-3′ to H-9′ and H-14′ to H-17′). ^13^C NMR (125 MHz, CDCl_3_): δ 176.4 (C-1), 170.0 (CH_3_COO), 139.1 (C-19′), 114.2 (C-20′), 80.2 (C-12′), 80.1 (C-11′), 77.3 (C-4), 72.4 (C-3), 45.6 (C-2), 33.7 (C-18′), 28.6–29.7 (C-3′ to C-9′ and C-14′ to C-17′), 27.3 (C-2′), 23.7 (C-1′), 20.4 (CH_3_COO), 18.7 (C-10′ and C-13′), 14.0 (CH_3_-4). ESI-HRMS (positive mode) *m/z* 455.3210 [M + Na]^+^ (calculated for C_27_H_44_O_4_Na 455.3137).

(2*S*,3*R*,4*R*)-3-Acetoxy-4-methyl-2-(*n*-eicos-11′-ynil)butanolide (**2a**). Amorphous white solid, 100% purity by HPLC. ^1^H NMR (500 MHz, CDCl_3_) δ 5.59 (dd, *J* = 5.3 and 3.2 Hz, H-3), 4.56 (dq, *J* = 6.4 and 3.2 Hz, H-4), 2.69 (dt, *J* = 9.7 and 5.2 Hz, H-2), 2.13 (t, *J* = 7.4 Hz, H-10′ and H-13′), 2.13 (s, CH_3_COO), 1.46 (m, H-1′), 1.32 (d, *J* = 6.4 Hz, CH_3_-4), 1.26 (br s, H-2′ to H-9′ and H-14′ to H-19′), 0.87 (t, *J* = 7.0 Hz, H-20′). ^13^C NMR (125 MHz, CDCl_3_) δ 176.4 (C-1), 170.0 (CH_3_COO), 80.2 (C-12′), 80.1 (C-11′), 77.3 (C-4), 72.4 (C-3), 45.6 (C-2), 31.8 (C-18′), 28.6–29.7 (C-3′ to C-9′ and C-14′ to C-17′), 27.3 (C-2′), 23.7 (C-1′), 22.7 (C-19′), 20.4 (CH_3_COO), 18.7 (C-10′ and C-13′), 14.1 (C-20′), 14.0 (CH_3_-4). ESI-HRMS (positive mode) *m/z* 457.3006 [M + Na]^+^ (calculated for C_27_H_46_O_4_Na 457.3294).

(2*S*,*3R*,4*R*)-3-Acetoxy-4-methyl-2-(*n*-eicosanil)butanolide (**3a**). Amorphous white solid, 100% purity by HPLC. ^1^H NMR (500 MHz, CDCl_3_) δ 5.59 (dd, *J* = 5.1 and 3.3 Hz, H-3), 4.55 (dq, *J* = 6.4 and 3.3 Hz, H-4), 2.69 (dt, *J* = 9.8 and 4.9 Hz, H-2), 2.12 (s, CH_3_COO), 1.82 (m, H-1′ and H-2′), 1.31 (d, *J* = 6.4 Hz, CH_3_-4), 1.25 (br s, H-3′ to H-9′), 0.87 (t, *J* = 6.2 Hz, H-20′). ^13^C NMR (125 MHz, CDCl_3_) δ 176.5 (C-1), 170.1 (CH_3_COO), 77.5 (C-4), 72.4 (C-3), 45.8 (C-2), 32.0 (C-18′), 29.8–29.4 (C-3′ to C-17′), 27.4 (C-2′), 23.8 (C-1′), 22.8 (C-19′), 20.5 (CH_3_COO), 14.2 (C-20′), 14.1 (CH_3_-4). ESI-HRMS (positive mode) *m/z* 461.3602 [M + Na]^+^ (calculated for C_27_H_50_O_4_Na 461.3607).

#### 3.4.3. Elimination (Compounds **1b**–**3b**)

Compound **3a** (30 mg) was placed on the top of an Al_2_O_3_ 90 (Merck, activity II/III, 10 g) column. Elution with pentane afforded **3b** (22 mg, 85%). This same procedure was performed using a mixture of compounds **1a** + **2a** (20 mg), and obtained products were purified by HPLC (eluent ACN:H_2_O 9:1, flow rate 1.0 mL/min, detection at 220 nm) to afford pure compounds **1b** (11 mg) and **2b** (4 mg). 

4*R*-Methyl-2-(*n*-eicos-11′-yn-19′-enil)but-2-enolide (**1b**). Amorphous white solid, 100% purity by HPLC. ^1^H NMR (500 MHz, CDCl_3_) δ 6.97 (d, *J* = 1.5 Hz, H-3), 5.80 (ddt, *J* = 16.9, 10.2 and 6.6 Hz, H-19′), 5.00 (m, H-4), 4.94 (m, H-20′), 2.26 (t, J = 7.3 Hz, H-1′), 2.13 (t, *J* = 6.8 Hz, H-10′ and H-13′), 2.04 (dt, *J* = 6.9 and 1.1 Hz, H-18′), 1.40 (d, *J* = 6.8 Hz, CH_3_-4), 1.27 (br s, H-2′ to H-9′ and H-14′ to H-17′). 13C NMR (125 MHz, CDCl_3_) δ 174.0 (C-1), 148.9 (C-3), 139.2 (C-19′),134.4 (C-2), 114.3 (C-20′), 80.4 (C-12′), 80.3 (C-11′), 77.5 (C-4), 33.8 (C-18′), 27.5 (C-2′), 25.3–29.6 (C-3′ to C-9′ and C-14′ to C-17′), 25.2 (C-1′), 18.8 (C-10′ and C-13′), 19.2 (CH_3_-4). ESI-HRMS (positive mode) *m/z* 395.2958 [M + Na]^+^ (calculated for C_25_H_40_O_2_Na 395.2926).

4*R*-Methyl-2-(*n*-eicos-11′-ynil)but-2-enolide (**2b**). Amorphous white solid, 100% purity by HPLC. ^1^H NMR (500 MHz, CDCl_3_) δ 6.97 (d, *J* = 1.5 Hz, H-3), 4.99 (m, H-4), 2.26 (t, *J* = 7,2 Hz, H-1′), 2.13 (t, *J* = 6.7 Hz, H-10′ and H-13′), 2.11 (d, *J* = 6.8 Hz, CH_3_-4), 1.27 (br s, H-2′ to H-9′ and H-14′ to H-19′), 0.87 (t, *J* = 6.9 Hz, H-20′). ^13^C NMR (125 MHz, CDCl_3_) δ 174.0 (C-1), 148.9 (C-3), 134.4 (C-2), 80.4 (C-12′), 80.3 (C-11′), 77.5 (C-4), 27.5 (C-2′), 25.3–29.6 (C-3′ to C-9′ and C-14′ to C-18′), 25.2 (C-1′), 22.7 (C-19′), 18.8 (C-10′ and C-13′), 19.2 (CH_3_-4), 18.8 (C-20). ESI-HRMS (positive mode) *m/z* 397.3118 [M + Na]^+^ (calculated for C_25_H_42_O_2_Na 397.3082).

4*R*-Methyl-2-(*n*-eicosanil)but-2-enolide (**3b**). Amorphous white solid, 100% purity by HPLC. ^1^H NMR (500 MHz, CDCl_3_) δ 6.97 (d, *J* = 1.5 Hz, H-3), 4.98 (m, H-4), 2.25 (t, *J* = 7,2 Hz, H-1′), 1.56 (m, H-2′), 1.40 (d, *J* = 6.8 Hz, CH_3_-4), 1.25 (br s, H-3′ to H-19′), 0.87 (t, *J* = 6.9 Hz, H-20′). ^13^C NMR (125 MHz, CDCl_3_) δ 173.9 (C-1), 148.8 (C-3), 134.3 (C-2), 77.4 (C-4), 31.9 (C-18′), 29.7–29.2 (C-3′ to C-17′), 27.4 (C-2′), 25.1 (C-1′), 22.7 (C-19′), 19.2 (C-20′), 14.1 (CH_3_-4). ESI-HRMS (positive mode) *m/z* 401.3387 [M + Na]^+^ (calculated for C_25_H_46_O_2_Na 401.3396).

### 3.5. In Silico Studies

In silico studies for bioactive compounds **1**, **1a**, and **1b** were conducted using the online platform SwissADME (available online: http://www.swissadme.ch/ (14 January 2022)). Using this approach, different aspects associated with pharmacokinetics, drug-likeness, and medicinal chemistry parameters were determined, including: ADME (Absorption, Distribution, Metabolism, and Excretion), physicochemical properties (number of rotatable bonds, number of H-bond donors and H-bond acceptors), lipophilicity (log *p* value), pharmacokinetics (gastrointestinal absorption and CYP 450 inhibitors), drug-likeness, especially Lipinski rule, and alert for PAINS (pan-assay interference compounds) [12].

### 3.6. Bioassay Procedures

#### 3.6.1. Animals

The animal breeding facility at the Instituto Adolfo Lutz, São Paulo, Brazil supplied the BALB/c mice and Golden hamsters (*Mesocricetus auratus*). The animals were maintained in sterilized cages under a controlled environment, receiving water and food ad libitum. Animal procedures were performed with the approval of the Research Ethics Commission, which is in agreement with the Guide for the Care and Use of Laboratory Animals from the National Academy of Sciences.

#### 3.6.2. Parasite Maintenance

*L. (L.) infantum* (MHOM/BR/1972/LD) was maintained through successive passages in golden hamsters up to 60–70 days post-infection. Amastigotes were harvested from spleens of infected hamsters by differential centrifugation [13].

#### 3.6.3. Mammalian Cells

Macrophages were collected from the peritoneal cavity of female BALB/c mice by washing with RPMI-1640 without phenol red, supplemented with 10% FBS at 37 °C in a humidified atmosphere containing 5% CO*_2_*. NCTC (clone 929) cells were maintained in RPMI-1640 (without phenol red and supplemented with 10% FBS) at 37 °C in the same conditions of the peritoneal macrophages [14].

#### 3.6.4. Determination of the Activity against *L. (L.) infantum*—Intracellular Amastigotes

The 50% inhibitory concentrations (IC_50_) against intracellular amastigotes for tested compounds were determined in infected macrophages. Peritoneal macrophages were obtained as described in the Section 3.6.3, and *L. (L.) infantum* amastigotes were obtained from spleens of infected hamsters by differential centrifugation. Peritoneal macrophages were seeded at 1 × 10^5^ cells per well in Nunc™ 16-well slide chambers (Aldrich, St. Louis, MO, USA) for 24 h at 37 °C in a 5% CO_2_-humidified incubator. Next, amastigotes were isolated from a spleen of an infected hamster as described [13], counted, seeded at a 1:10 macrophages/amastigotes ratio, and incubated for 24 h. Non-internalized parasites were removed by washing twice with a culture medium. Then, the macrophages were incubated with compounds or standard drug (miltefosine) in a range of 100 to 0.78 μM for 96 h at 37 °C in a 5% CO_2_-humidified incubator, using miltefosine as a standard drug. At the end of the assay, the cells were fixed in methanol, stained with Giemsa, and observed under a digital light microscope (EVOS M5000, Thermo, Waltham, MA, USA) to determine the number of infected macrophages out of 400 cells [8].

#### 3.6.5. Determination of the Cytotoxicity against Mammalian Cells

The 50% cytotoxic concentration (CC_50_) was determined in NCTC clone 929 cells. NCTC cells were seeded at 6 × 10^4^ cells/well in 96-well microplates and incubated with serial dilutions of the tested compounds or standard drug (miltefosine) to the highest concentration of 200 μM for 48 h at 37 °C in a 5% CO_2_-humidified incubator. An MTT assay determined the viability of the cells at 570 nm [15]. The selectivity index (SI) was determined considering the following equation: CC_50_ NCTC cells/IC_50_ parasites.

#### 3.6.6. Statistical Analysis

The data obtained represent the mean and standard deviation of duplicate samples from at least three independent assays. IC_50_ and CC_50_ values were calculated using sigmoid dose–response curves in Graph Pad Prism 5.0 software (GraphPad Software, San Diego, CA, USA).

## 4. Conclusions

This work reports the isolation and antileishmanial activity of two natural acetogenins isolated from seeds of *P. macrocarpa* (**1** and **2**) and seven chemically related derivatives (**1a**, **1b**, **2a**, **2b**, **3**, **3a**, and **3b**). Among these compounds, **1** and **1b** exhibited more substantial potential, with similar IC_50_ values of positive control (miltefosine) and reduced mammalian cytotoxicity. However, chemically related compounds **2**, **2a**, **2b**, **3**, **3a**, and **3b** showed no activity, indicating that a double bond at C-19 and a triple bond at C-11 are crucial to the antileishmanial effect. However, the effect observed to the mixture of natural acetogenins **1** and **2** at 2:1 proportion showed superior selectivity to those determined to the isolated compounds. Therefore, our data corroborate the promising activity of acetylene derivatives from *P. macrocarpa* as antiprotozoal agents, especially for the mixture of natural acetogenins **1** and **2**.

## Figures and Tables

**Table 1:** Antileishmanial activity (amastigotes of *L. (L.) infantum*) and cytotoxic effects (NCTC cells) of natural compounds **1**–**3**, semisynthetic derivatives **1a**–**3a**, **1b**–**3b**, and standard drug (miltefosine).

Compound	IC_50_/μM *L. (L.) Infantum*	CC_50_/μM NCTC	SI
**1**	29.9 ± 9.7	>200	>6.7
**2**	NA	>200	-
mixtures of 1 and 2 *			
2:1	8.4 ± 3.6	>200	>23.8
1:1	13.6 ± 4.3	>200	>14.4
1:2	19.4 ± 7.8	>200	>10.3
**1a**	43.4 ± 3.9	>200	>4.6
**2a**	NA	>200	-
mixtures of 1a and 2a *			
2:1	12.0 ± 2.0	>200	>16.7
1:1	23.1 ± 6.5	>200	>8.7
1:2	38.4 ± 6.2	>200	>5.2
**1b**	23.1 ± 5.4	>200	>8.6
**2b**	NA	>200	-
mixtures of 1b and 2b *			
2:1	7.9 ± 4.4	>200	>25.3
1:1	10.5 ± 7.1	>200	>19.0
1:2	18.2 ± 9.0	>200	>11.0
**3**	NA	>200	-
**3a**	NA	>200	-
**3b**	NA	>200	-
**Miltefosine**	17.8 ± 1.4	116.0 ± 5.3	6.5

* mixtures were prepared using w:w proportion. NA: non-active. SI: selectivity-index.

**Table 2:** Physicochemical properties and ADMET predictions for compounds **1, 1a**, and **1b**.

Physicochemical Properties	1	1a	1b
Num. heavy atoms	28	31	27
Fraction Csp^3^	0.77	0.78	0.72
Num. rotatable bonds	16	18	16
Num. H-bond acceptors	2	4	2
Num. H-bond donors	1	0	0
log *P*_o/w_ (iLOGP)	6.15	5.64	5.80
Water Solubility	Poorly	Poorly	Poorly
GI absorption	Low	Low	Low
BBB permeant	No	No	No
CYP1A2 inhibitor	Yes	Yes	Yes
CYP2C19 inhibitor	No	No	No
CYP2C9 inhibitor	No	Yes	Yes
CYP2D6 inhibitor	No	No	No
CYP3A4 inhibitor	No	No	No
Lipinski	One violation (log *p* > 4.15)	One violation (log *p* > 4.15)	One violation (log *p* > 4.15)
Bioavailability Score	0.55	0.55	0.55
PAINS alert	No	No	No

## Data Availability

Not applicable.

## Supplementary Materials

The following supporting information can be downloaded online, Figures S1–S3: NMR and MS spectra of compound **1**, Figures S4–S6: NMR and MS spectra of compound **2**, Figure S7–S9: NMR and MS spectra of compound **1a**, Figure S10–S12: NMR and MS spectra of compound **1b**, Figure S13–S15: NMR and MS spectra of compound **2a**; Figure S16–S18: NMR and MS spectra of compound **2b**; Figure S19–S21: NMR and MS spectra of compound **3**; Figure S22–S24: NMR and MS spectra of compound **3a**; Figure S25–S27: NMR and MS spectra of compound **3b**.

## Sample Availability

Samples of tested compounds are available from the authors.
