# Supplementary material for: Antileishmanial Effects of Acetylene Acetogenins from Seeds of Porcelia macrocarpa (Warm.) R.E. Fries (Annonaceae) and Semisynthetic Derivatives
Source: Molecules. 2022 Jan 28;27(3):893. doi: 10.3390/molecules27030893 (PMC8838408; doi:10.3390/molecules27030893)

Figure S1: ^{1}H NMR spectrum (δ, $CDCl_3$, 300 MHz) of compound **1**

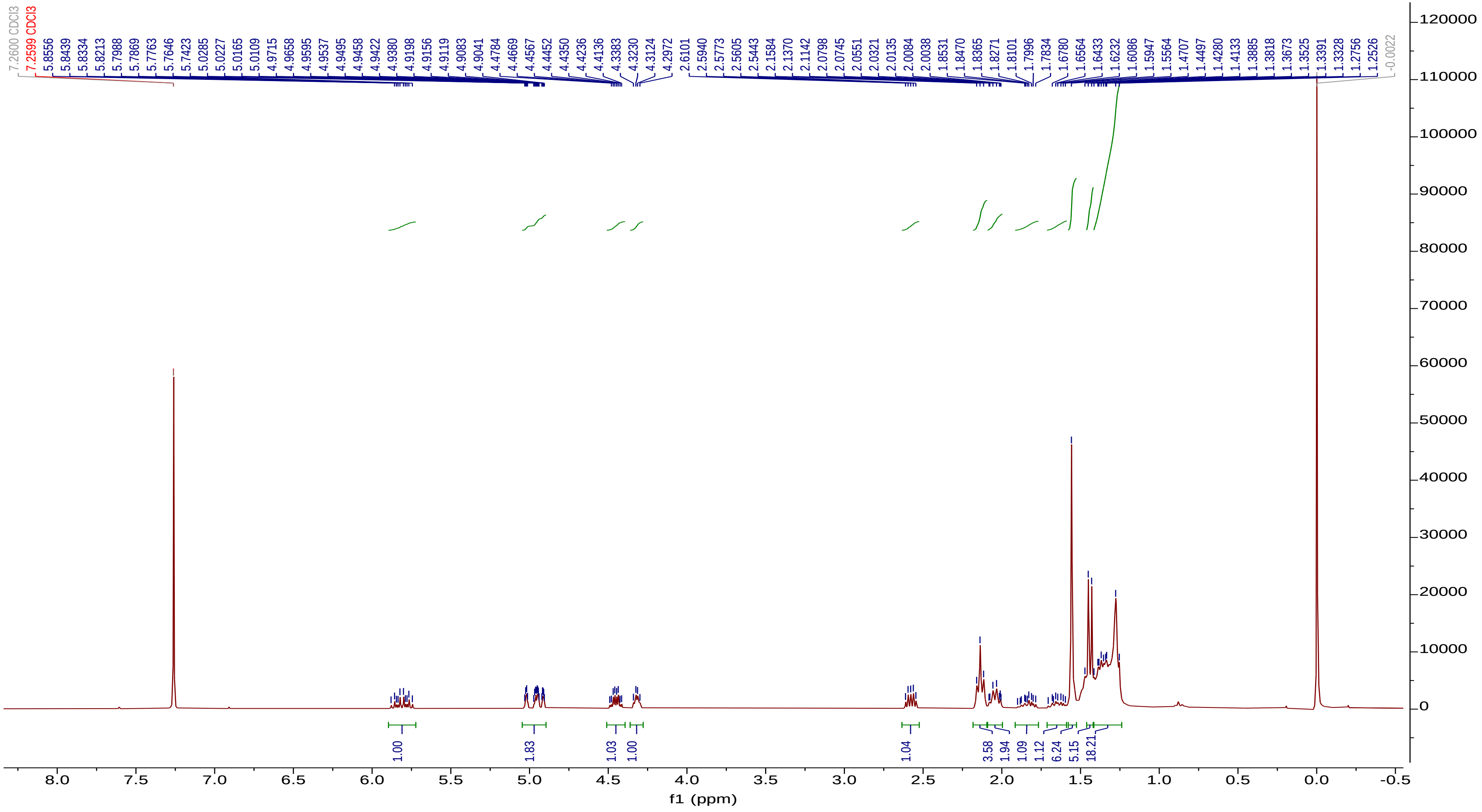

Figure S2: ^{13}C NMR spectrum (δ, $CDCl_3$, 125 MHz) of compound **1**

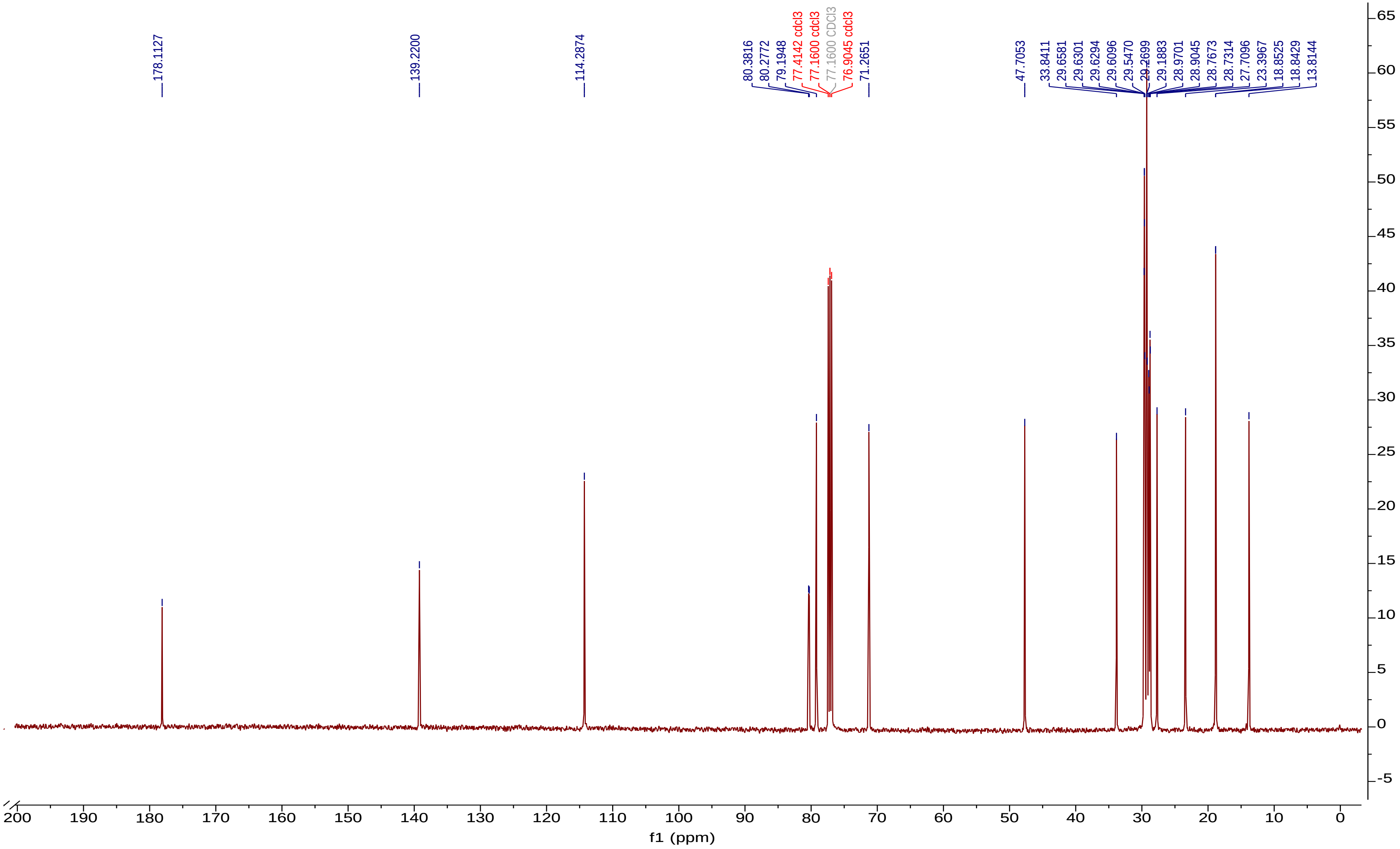

Figure S3: Mass spectrum (APCI - positive mode) - compound **1**

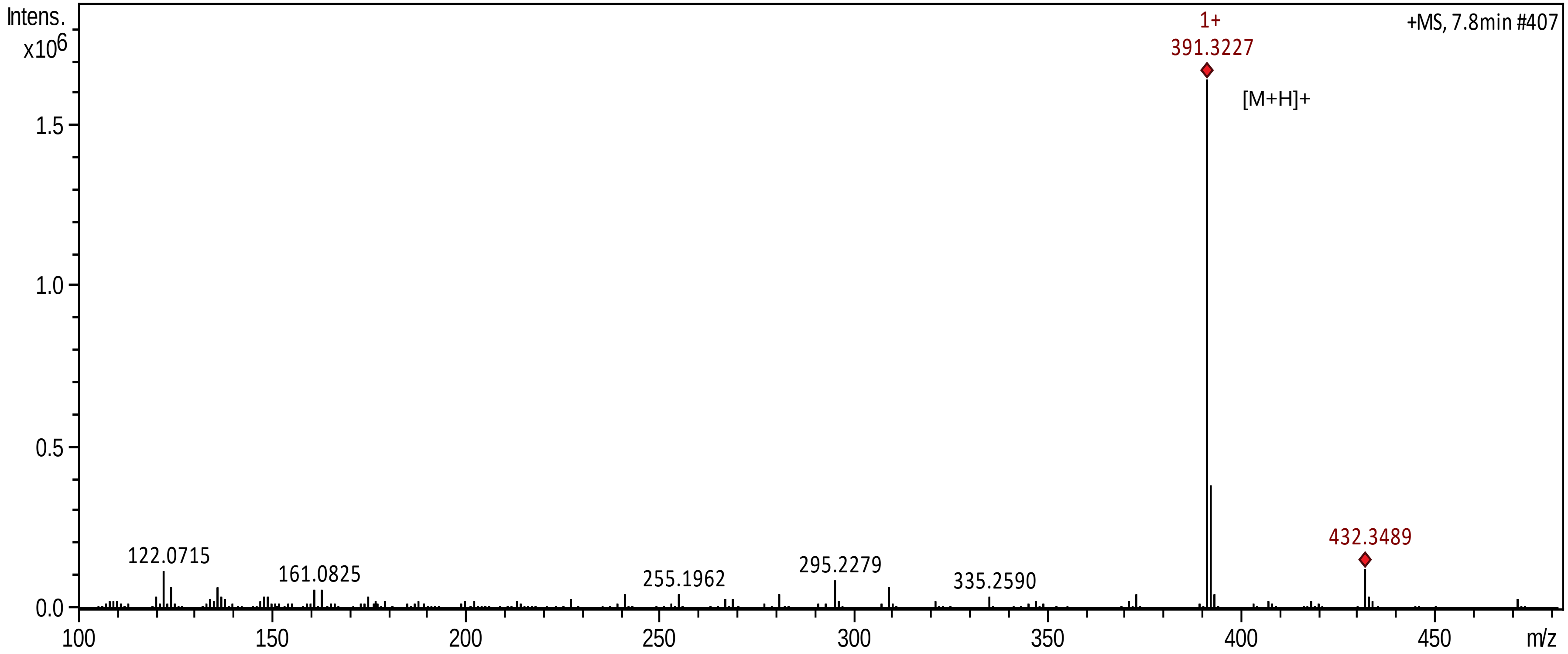

Figure S4: ^{1}H NMR spectrum (δ, $CDCl_3$, 300 MHz) of compound **2**

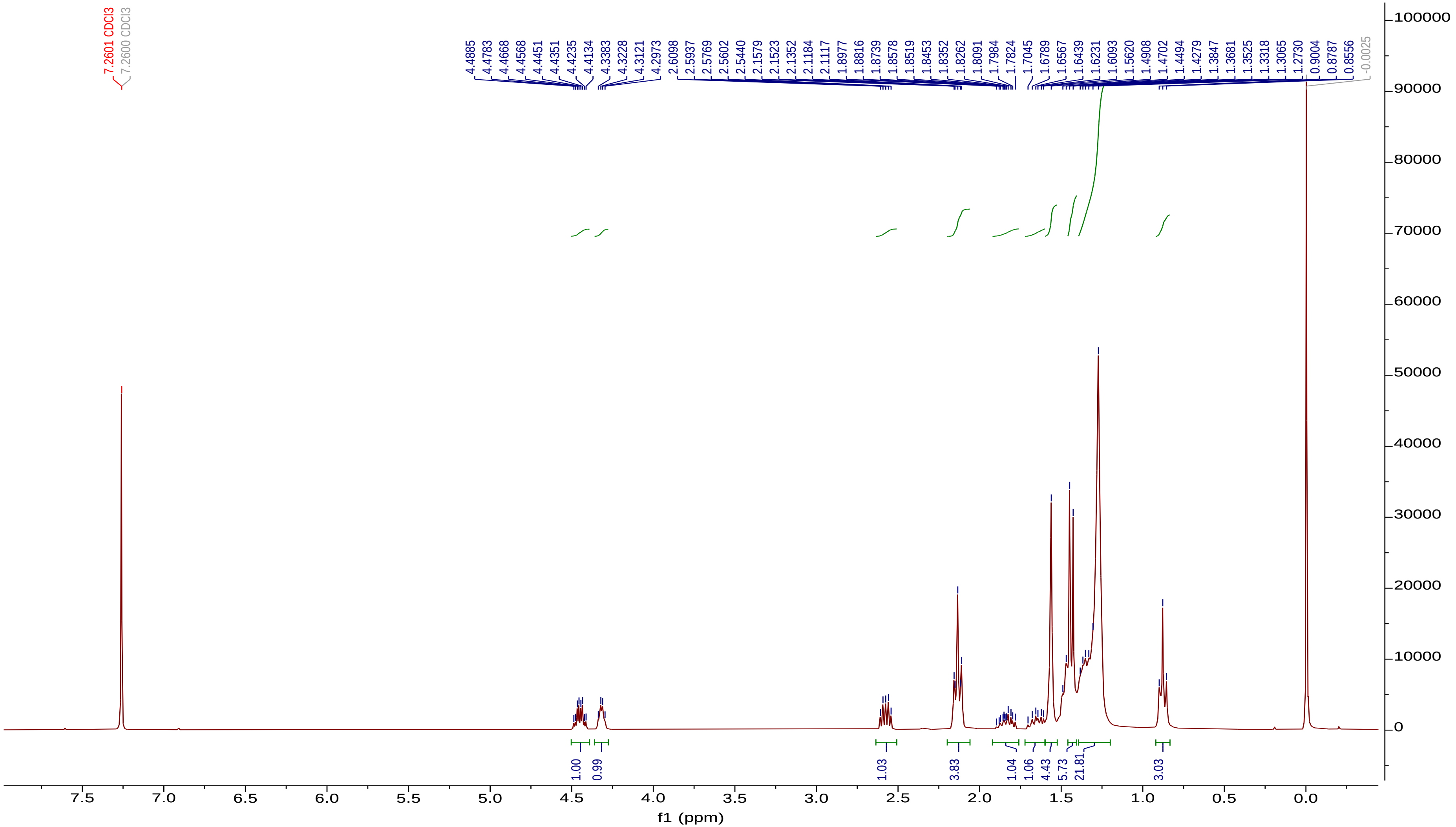

Figure S5: ^{13}C NMR spectrum (δ, $CDCl_3$, 125 MHz) of compound **2**

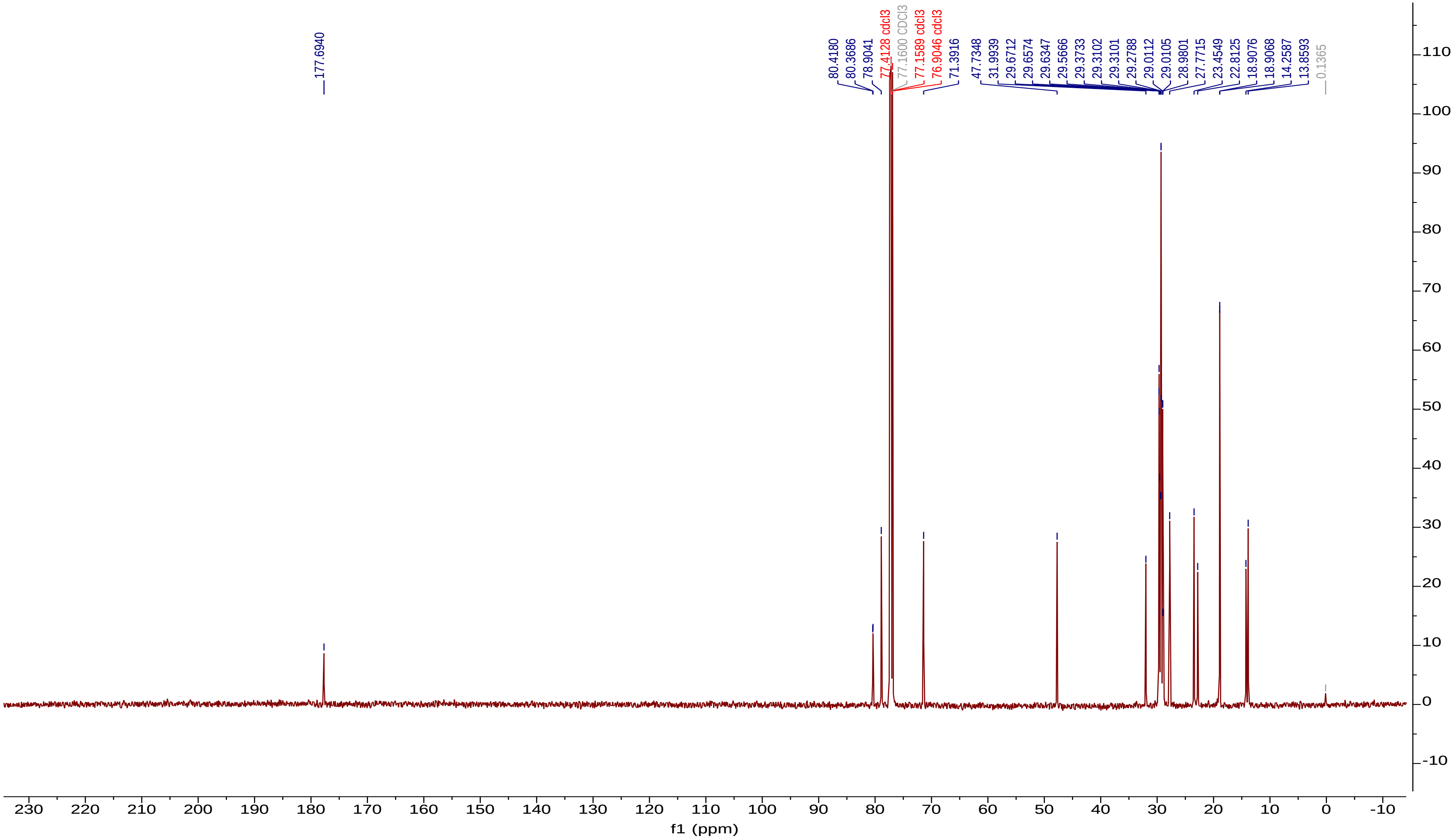

Figure S6: Mass spectrum (APCI - positive mode) - compound **2**

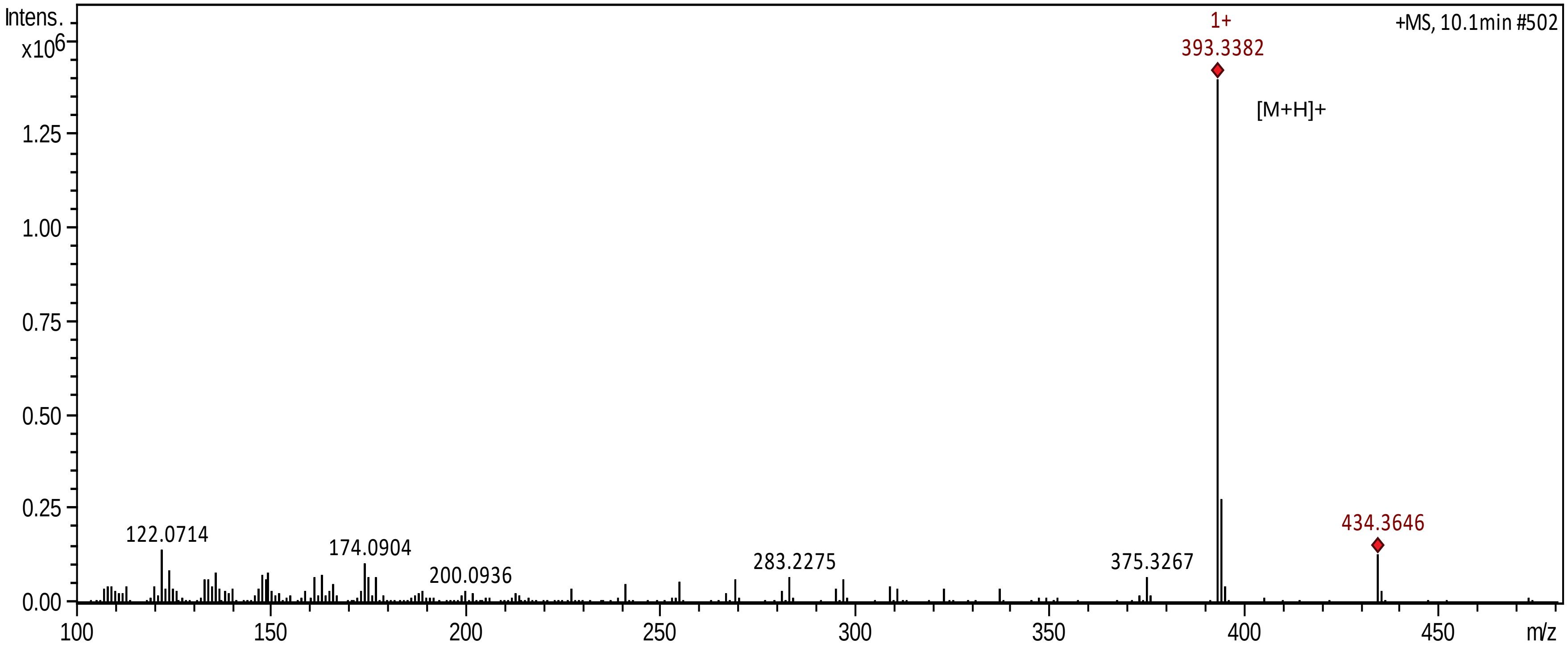

Figure S7: ^{1}H NMR spectrum (δ, $CDCl_3$, 300 MHz) of compound **1a**

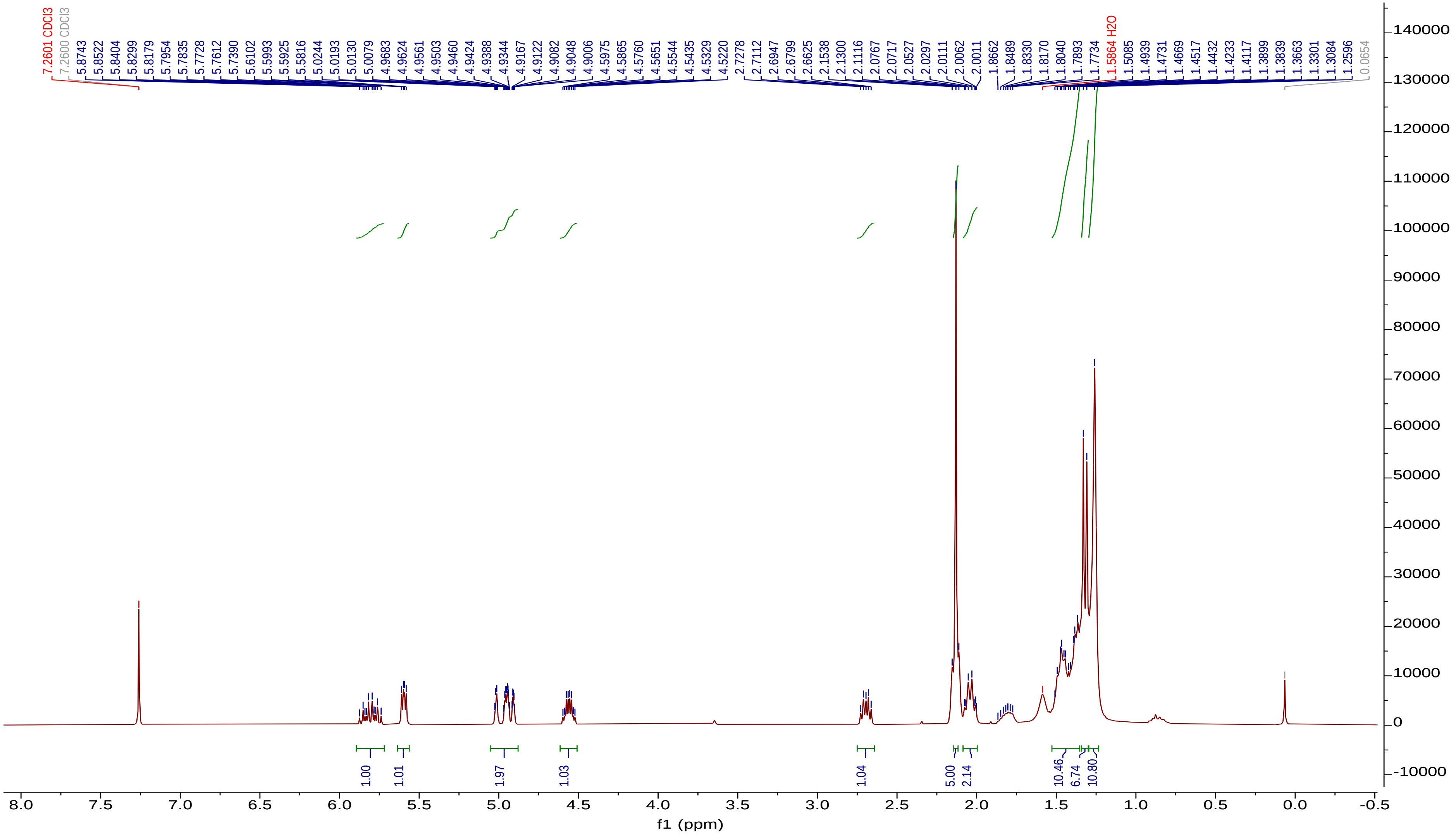

Figure S8: ^{13}C NMR spectrum (δ, $CDCl_3$, 125 MHz) of the mixture of compounds **1a** and **2a**

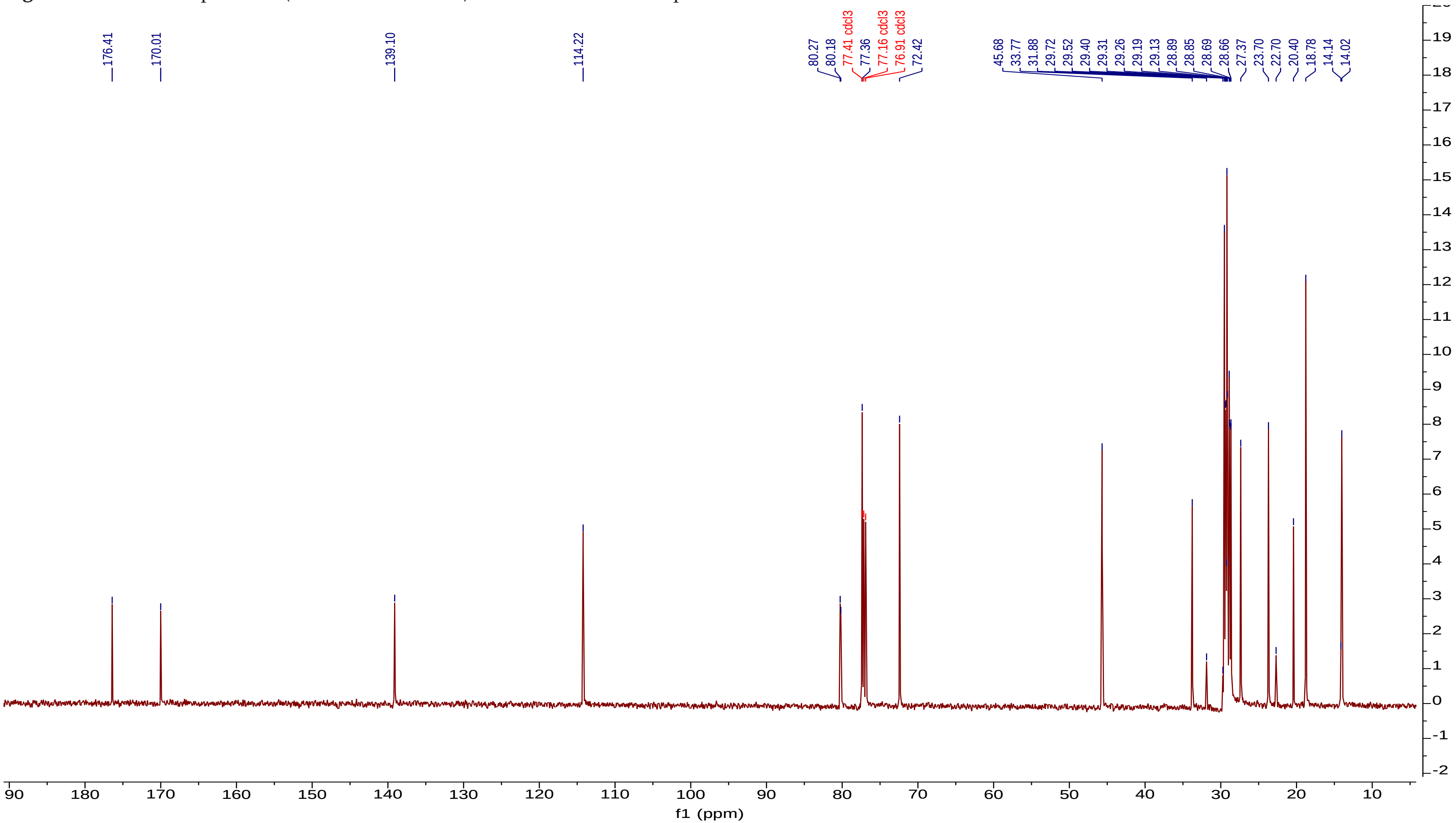

Figure S9: Mass spectrum (ESI - positive mode) - compound **1a**

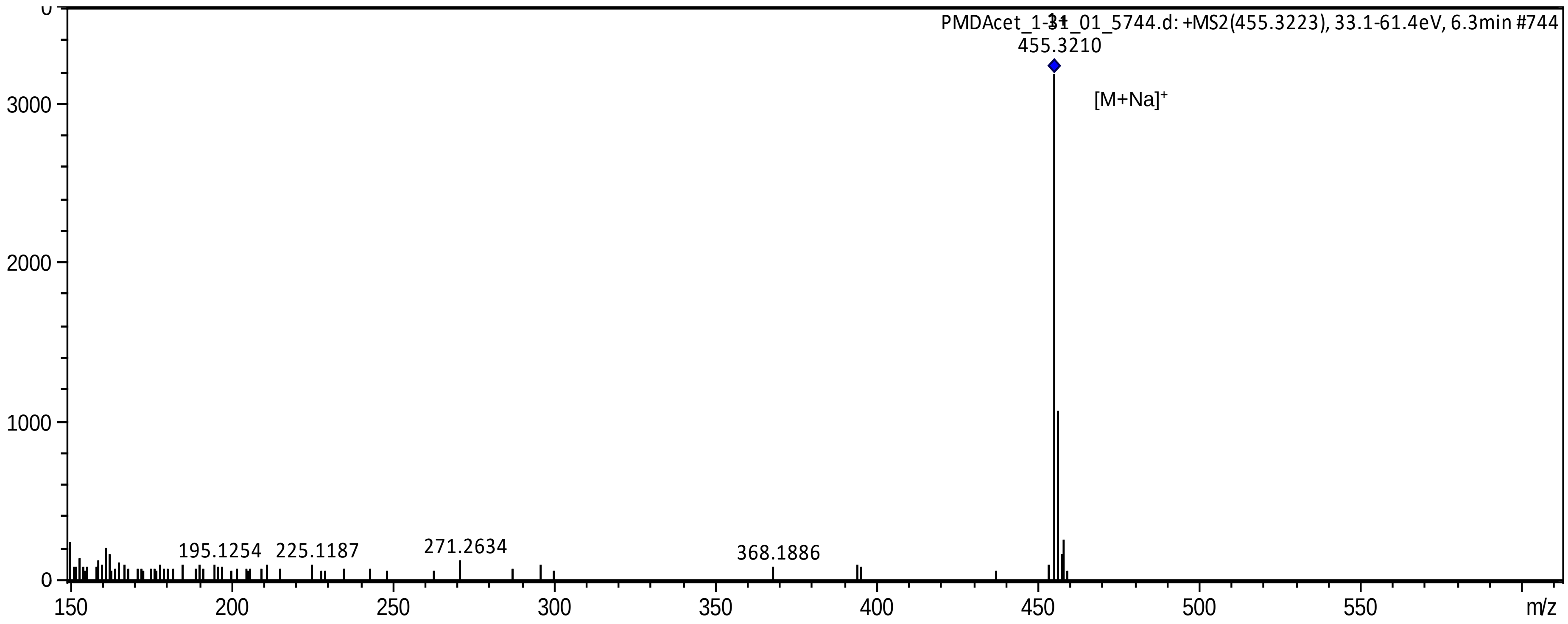

Figure S10: ^{1}H NMR spectrum (δ, $CDCl_3$, 300 MHz) of compound **1b**

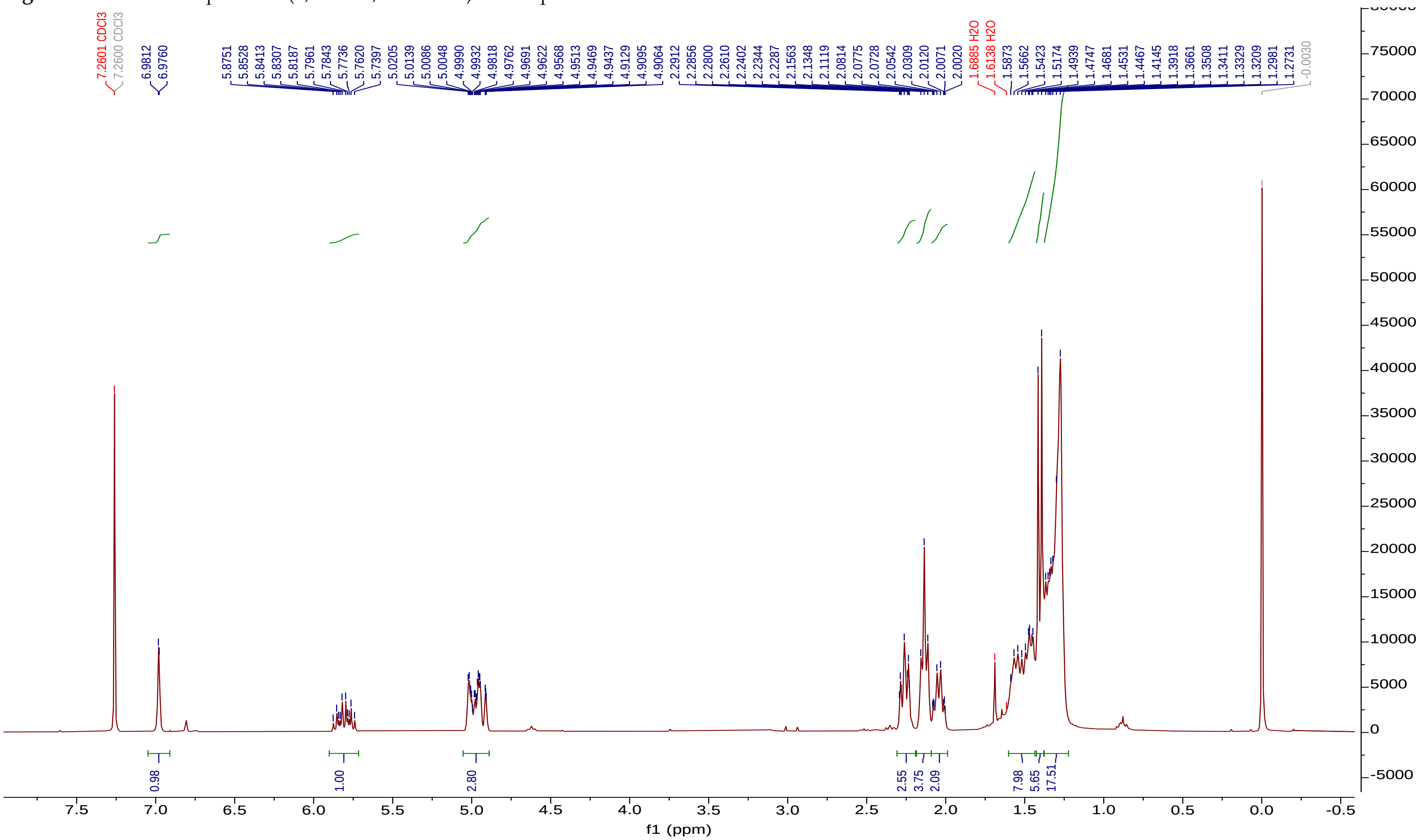

Figure S11: ^{13}C NMR spectrum (δ, $CDCl_3$, 125 MHz) of the mixture of compounds **1b** and **2b**

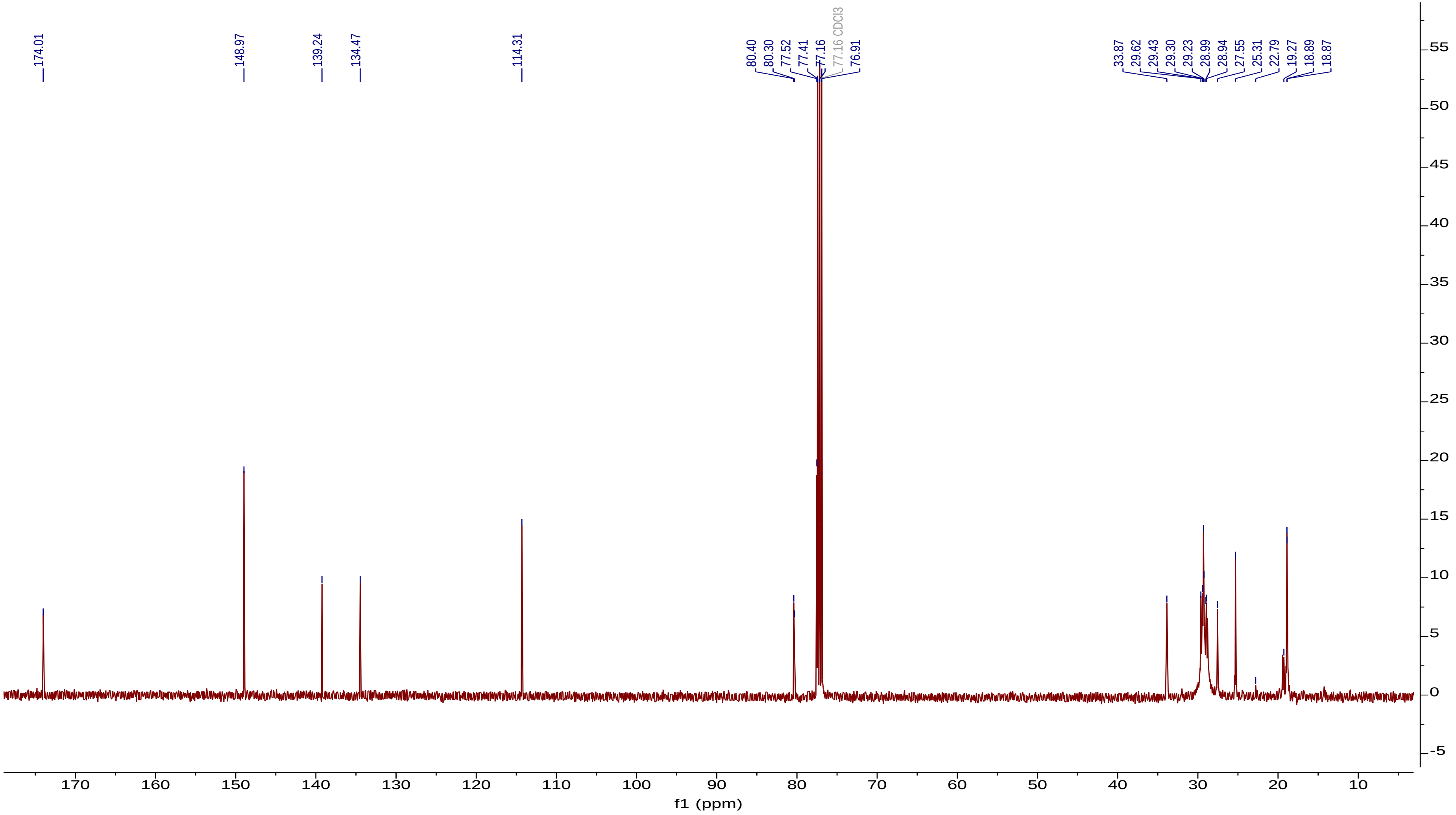

Figure S12: Mass spectrum (ESI - positive mode) – compound **1b**

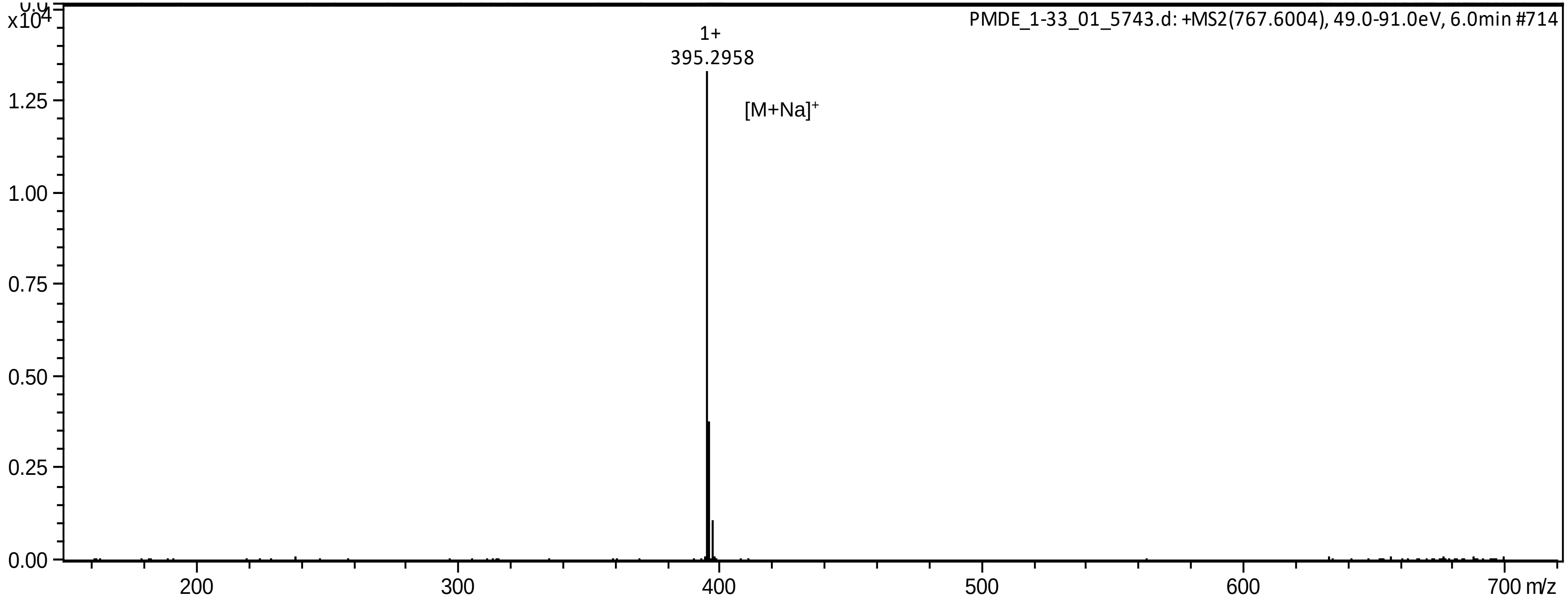

Figure S13: ^{1}H NMR spectrum (δ, $CDCl_3$, 300 MHz) of compound **2a**

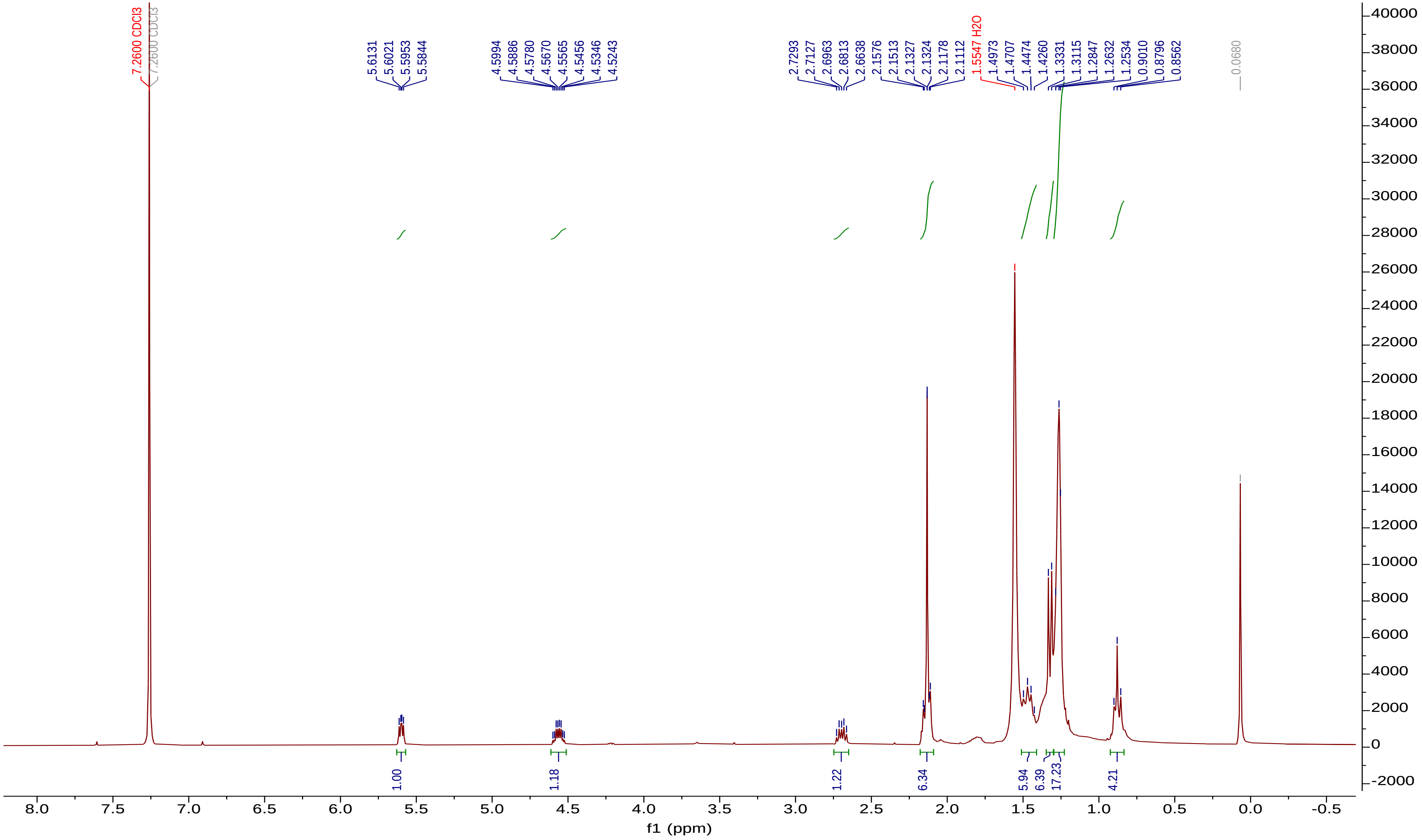

Figure S14: ^{13}C NMR spectrum (δ, $CDCl_3$, 125 MHz) of the mixture of compounds **1a** and **2a**

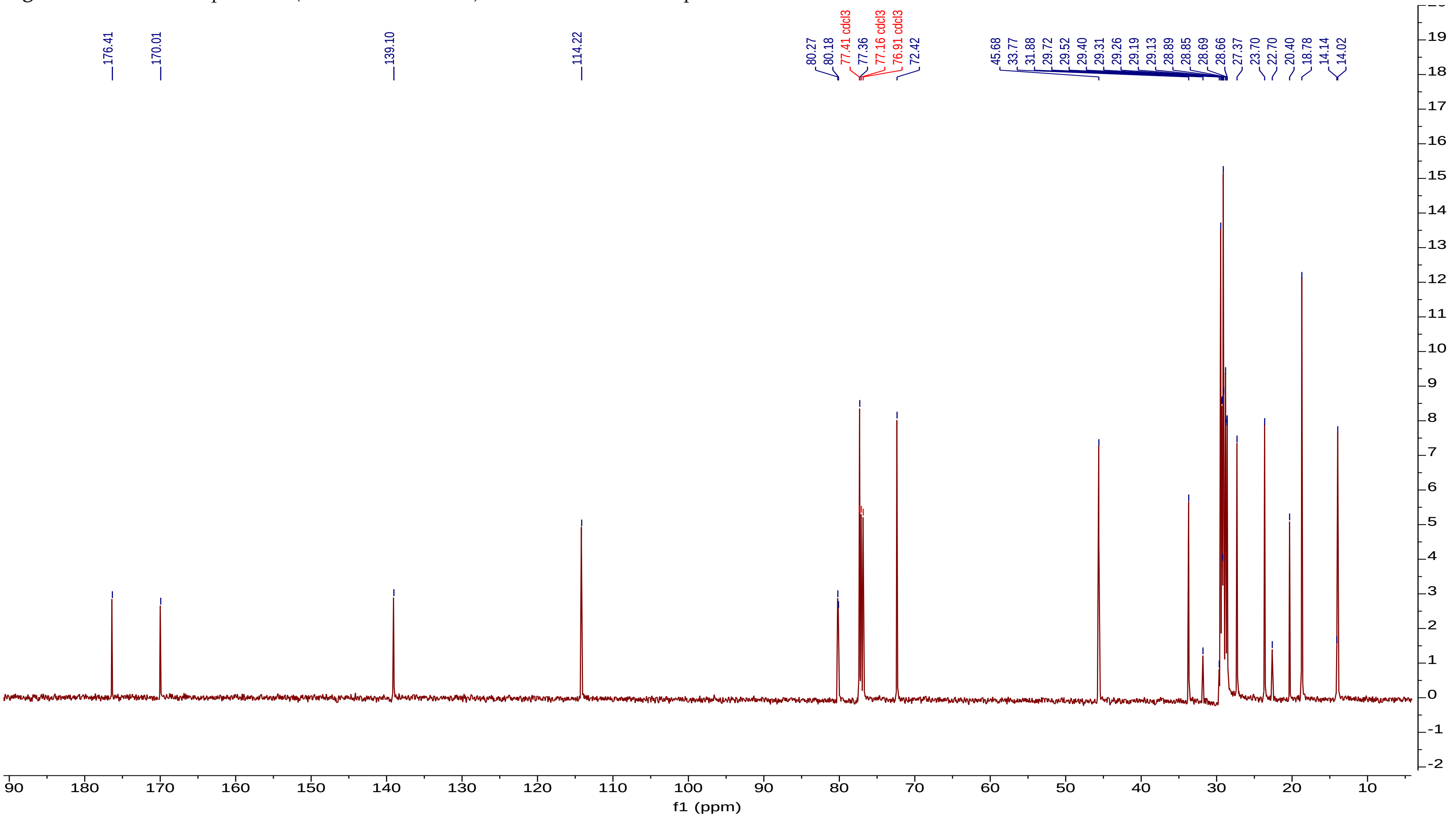

Figure S15: Mass spectrum (ESI - positive mode) - compound **2a**

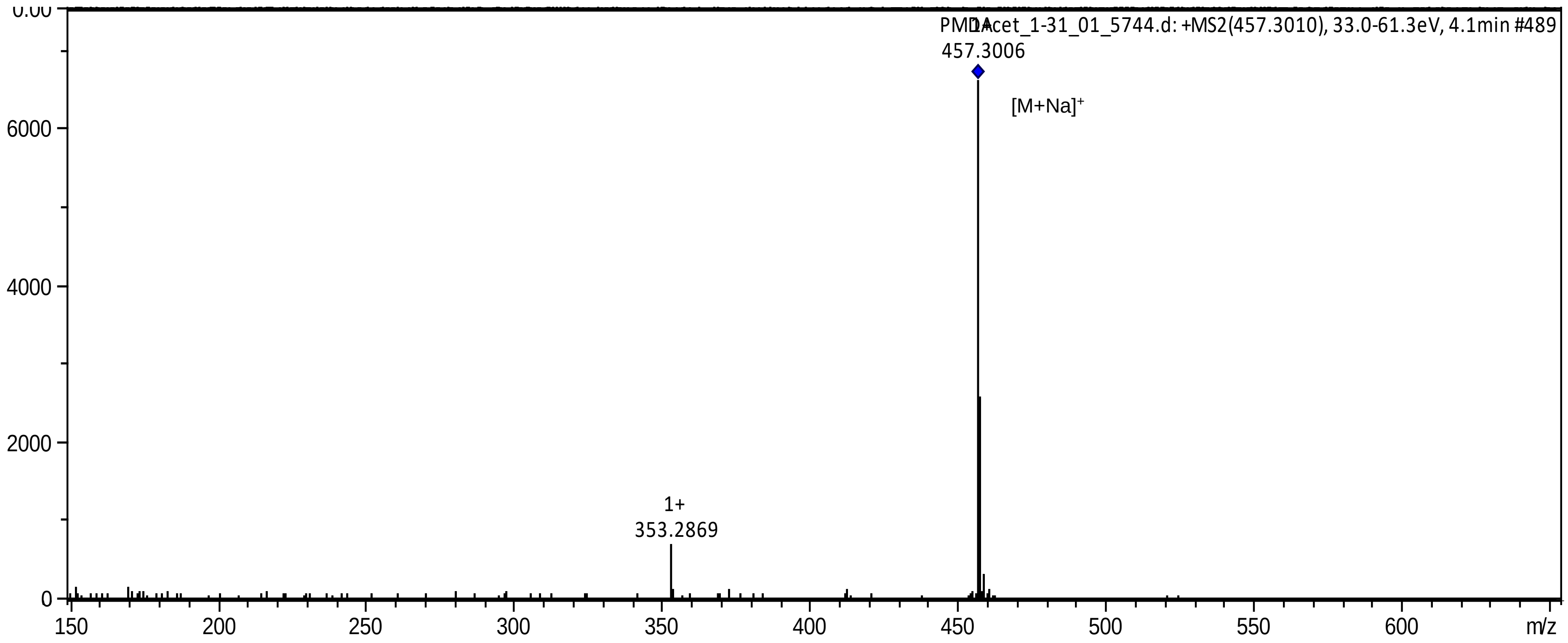

Figure S16: ^{1}H NMR spectrum (δ, $CDCl_3$, 300 MHz) of compound **2b**

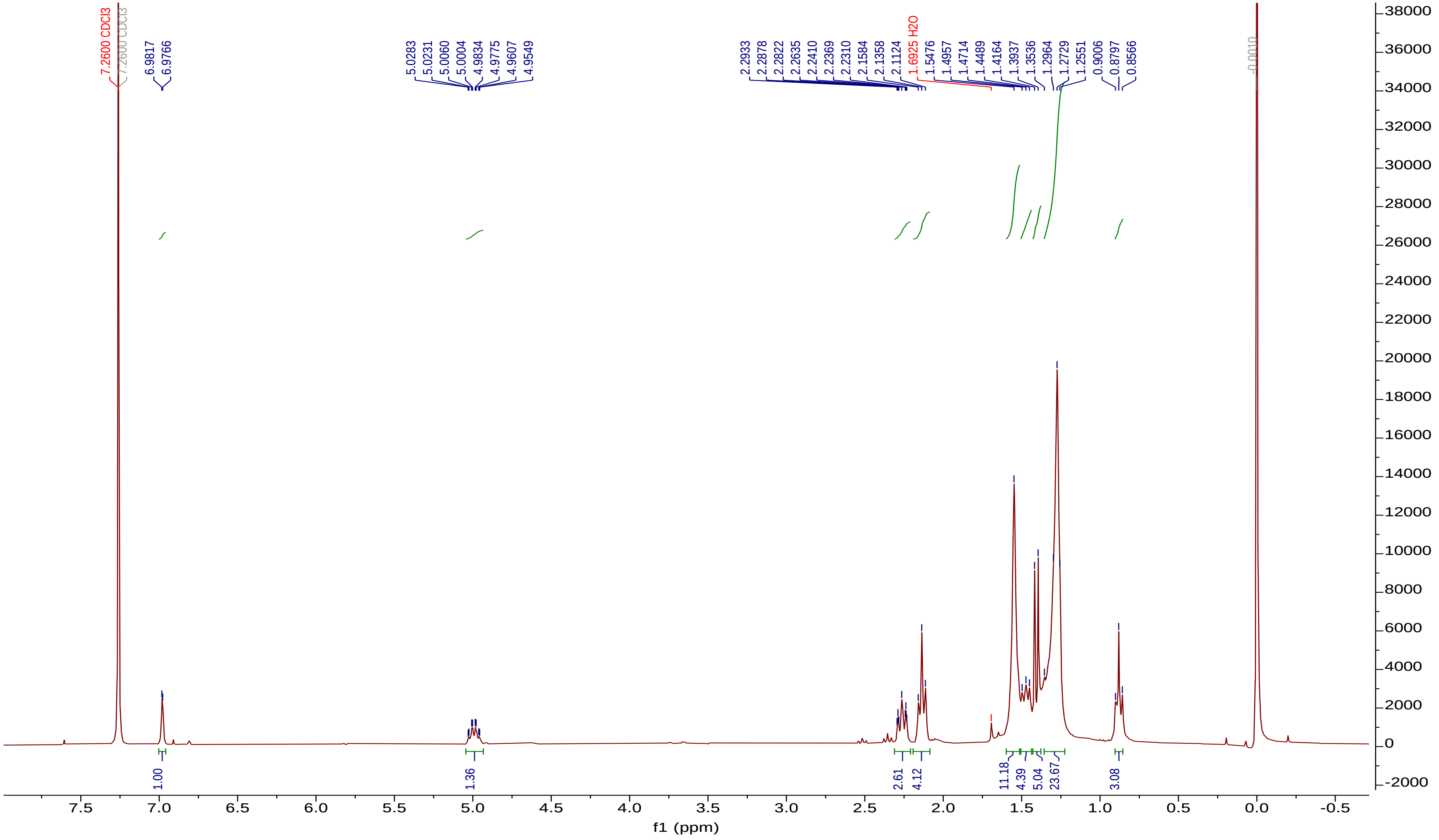

Figure S17: ^{13}C NMR spectrum (δ, $CDCl_3$, 125 MHz) of the mixture of compounds **1b** and **2b**

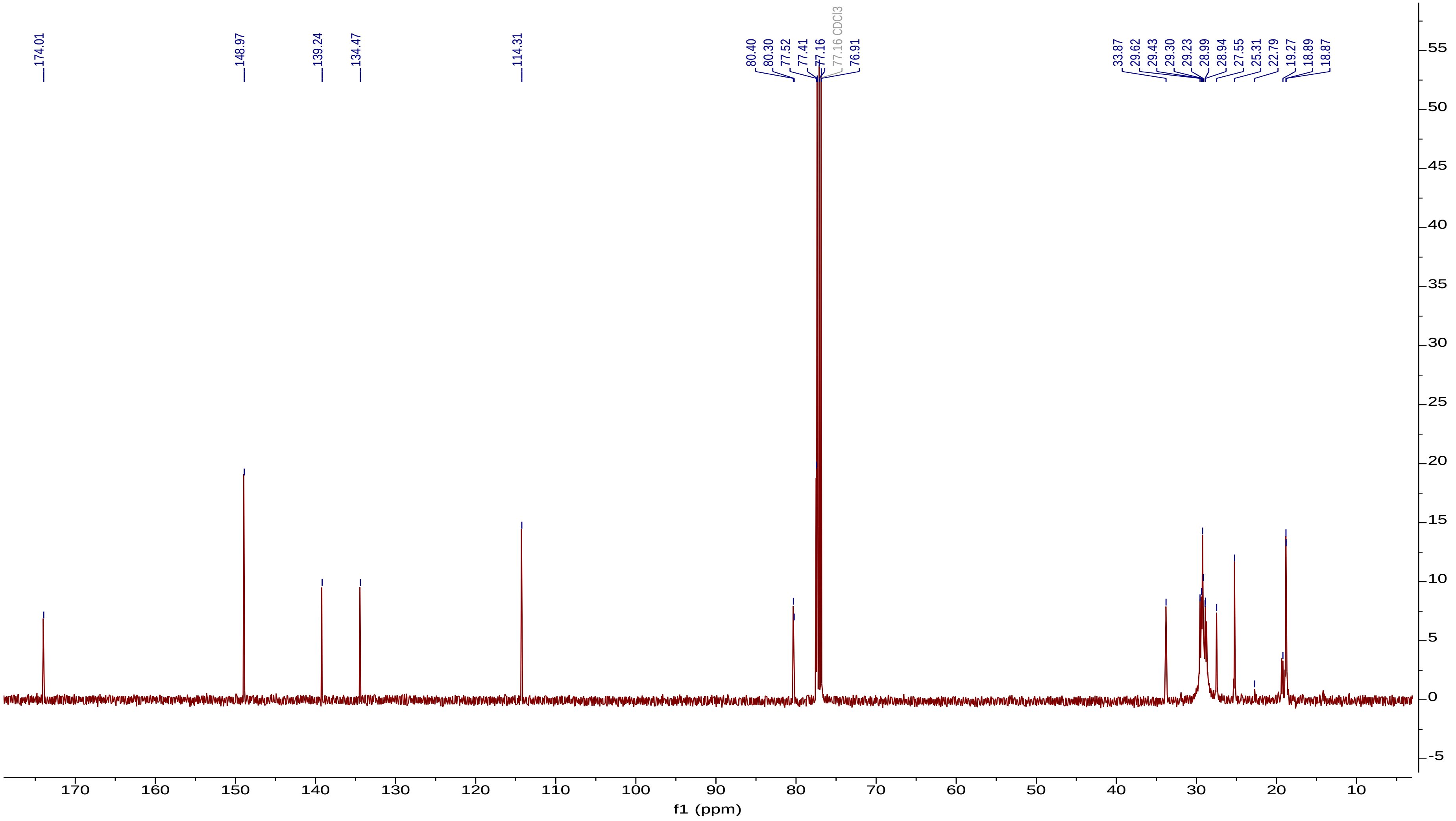

Figure S18: Mass spectrum (ESI - positive mode) – compound **2b**

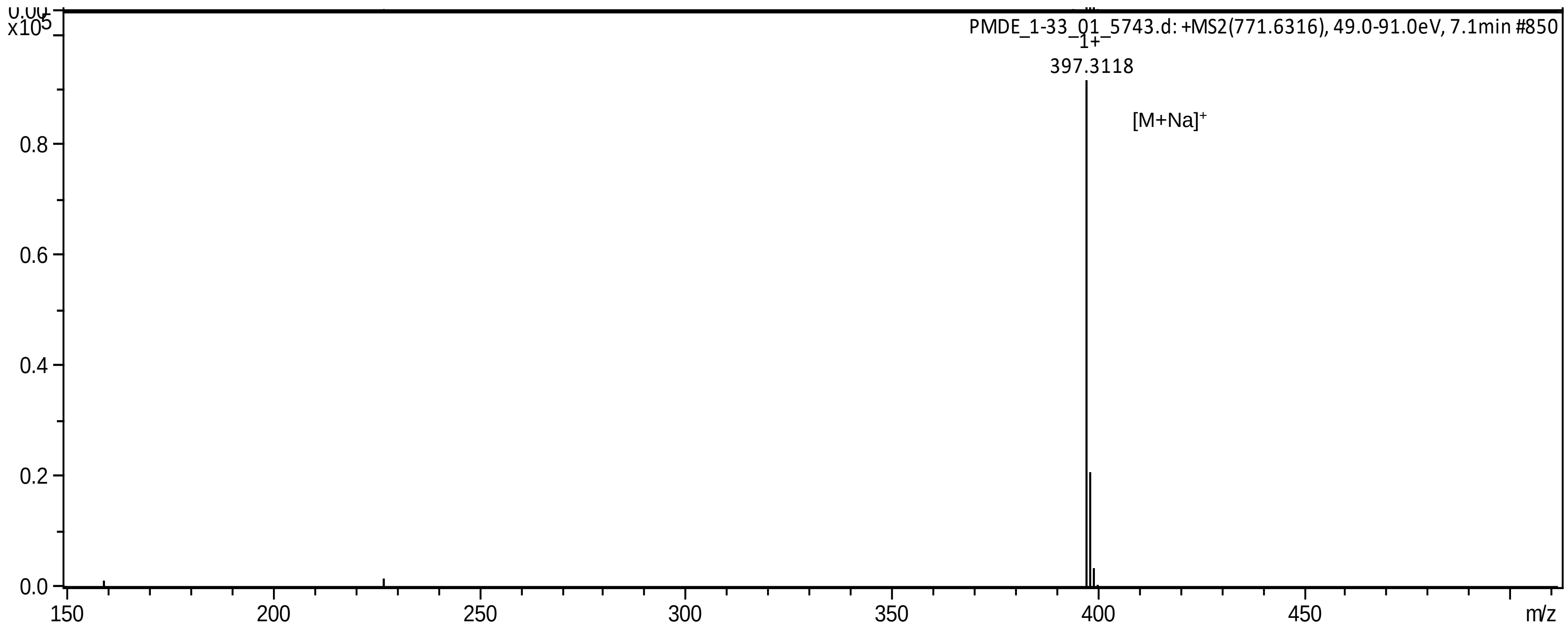

Figure S19: ^{1}H NMR spectrum (δ, $CDCl_3$, 500 MHz) of compound **3**

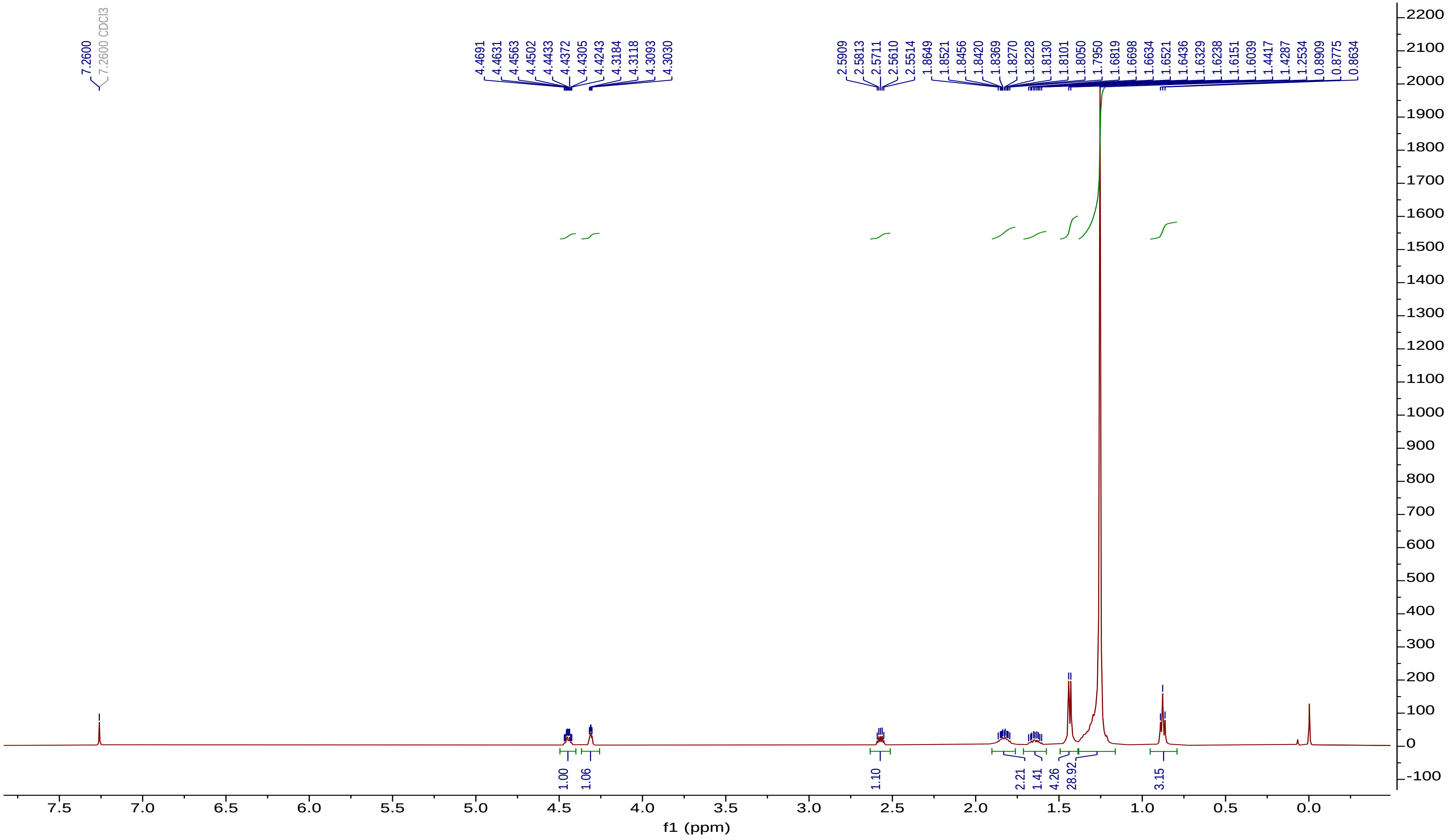

Figure S20: ^{13}C NMR spectrum (δ, $CDCl_3$, 125 MHz) of compound **3**

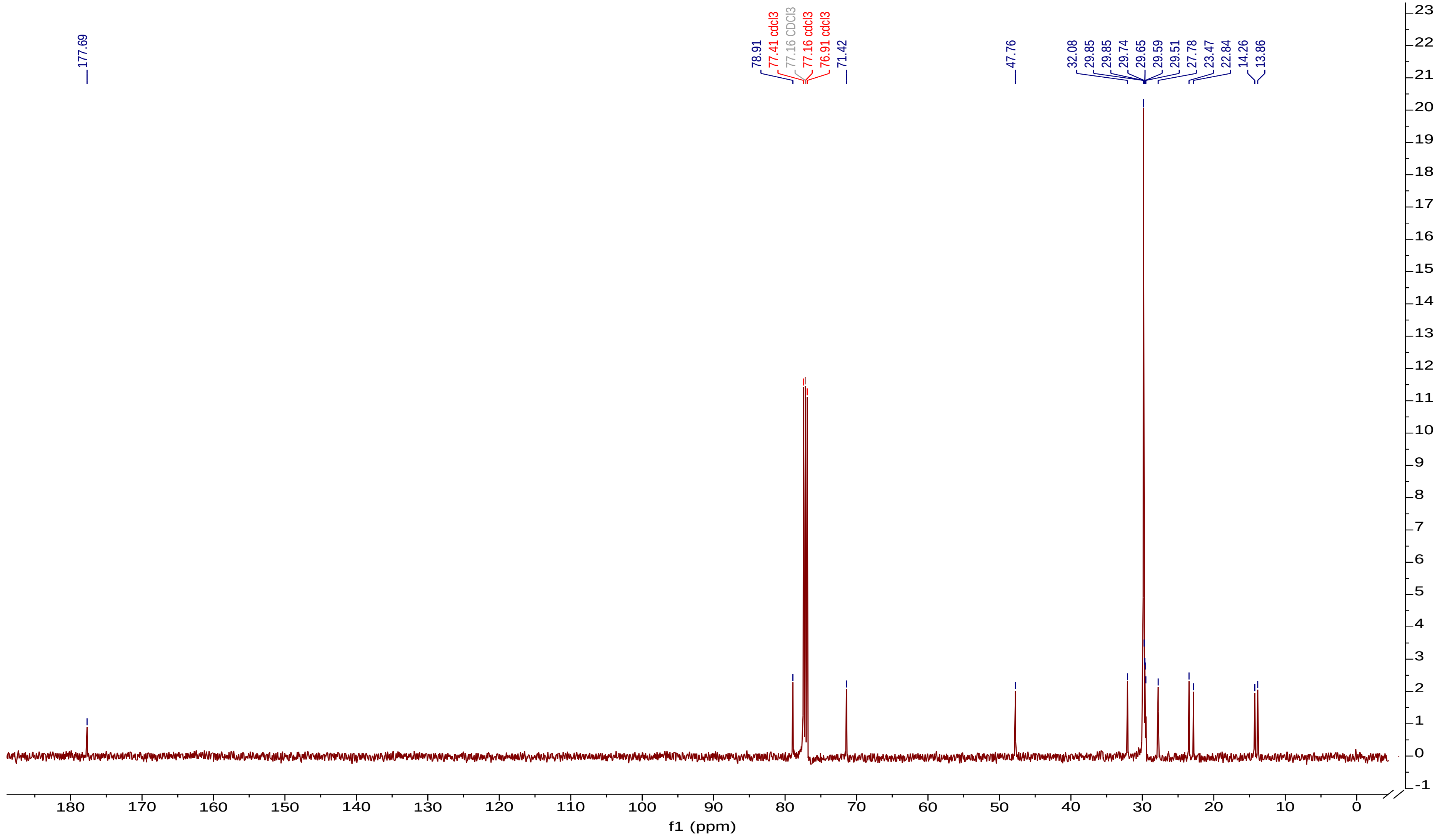

Figure S21: Mass spectrum (ESI - positive mode) - compound **3**

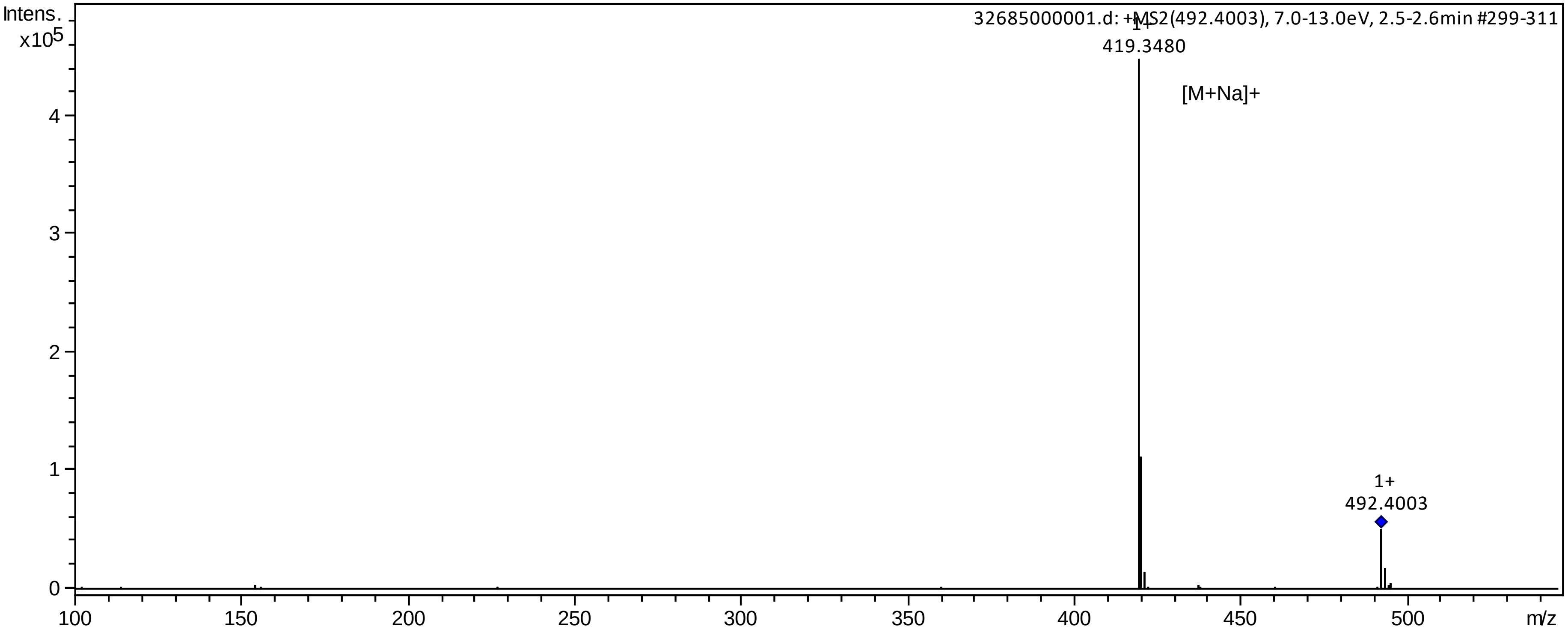

Figure S22: ^{1}H NMR spectrum (δ, $CDCl_3$, 300 MHz) of compound **3a**

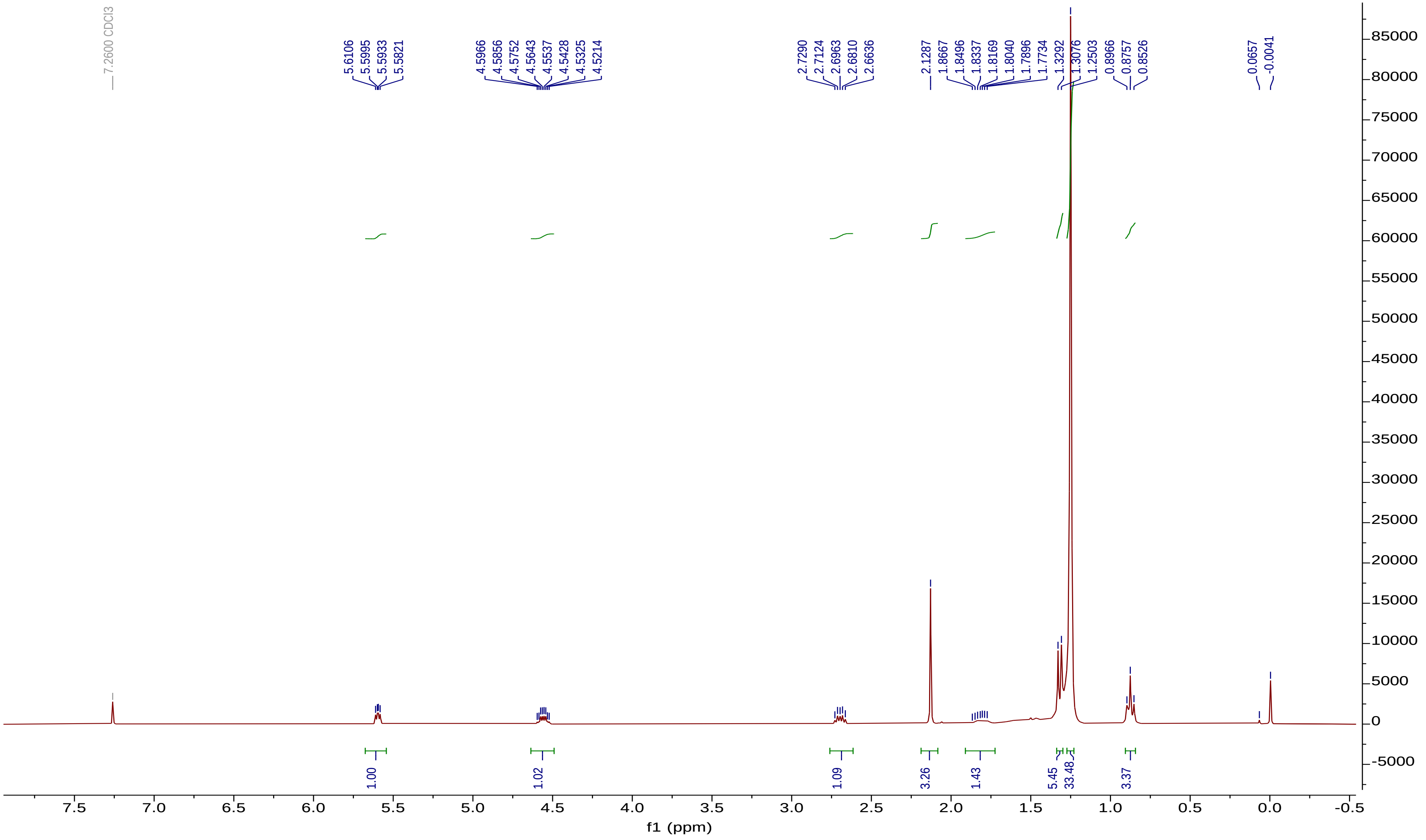

Figure S23: ^{13}C NMR spectrum (δ, $CDCl_3$, 75 MHz) of compound **3a**

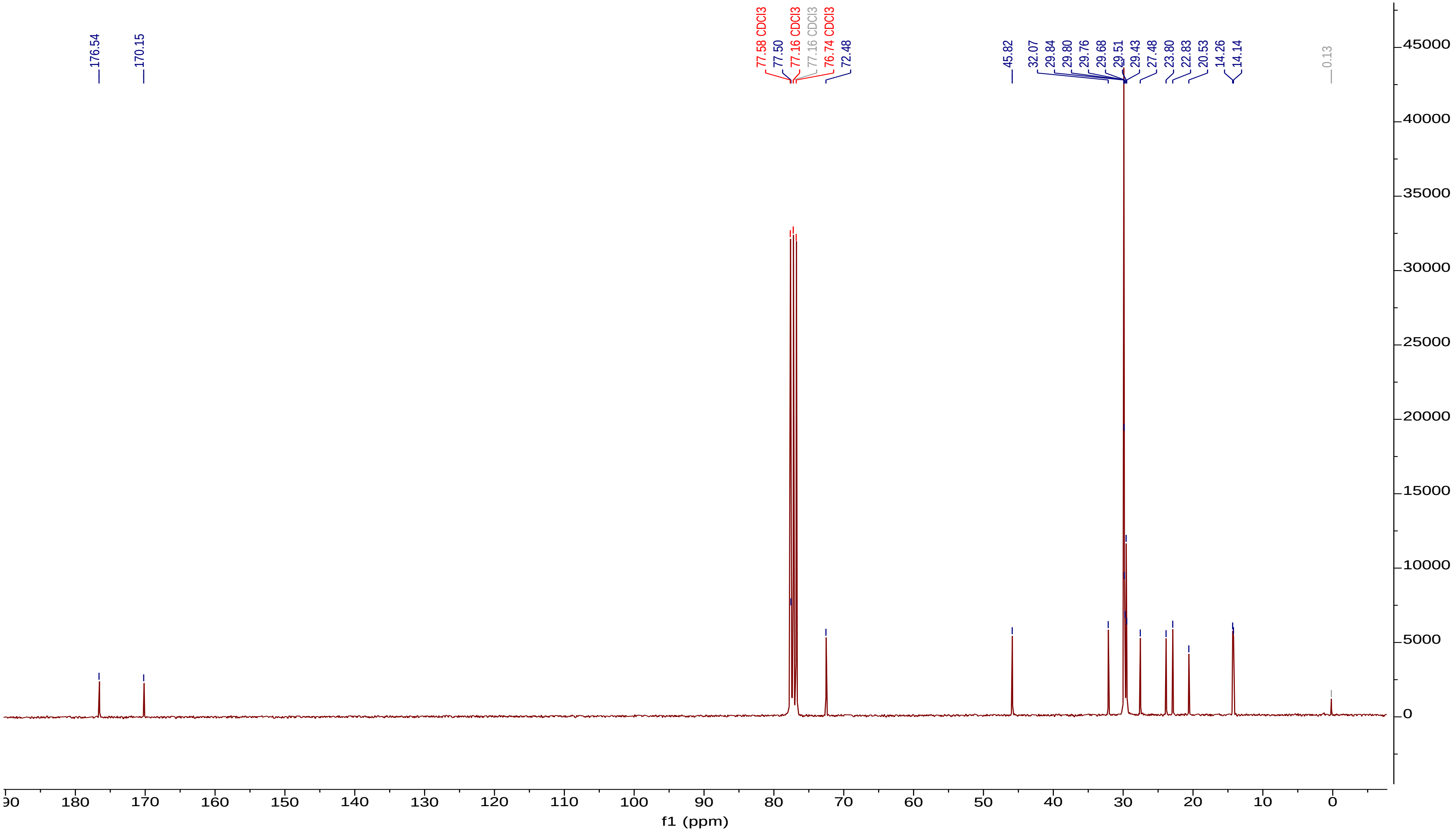

Figure S24: Mass spectrum (ESI - positive mode) - compound **3a**

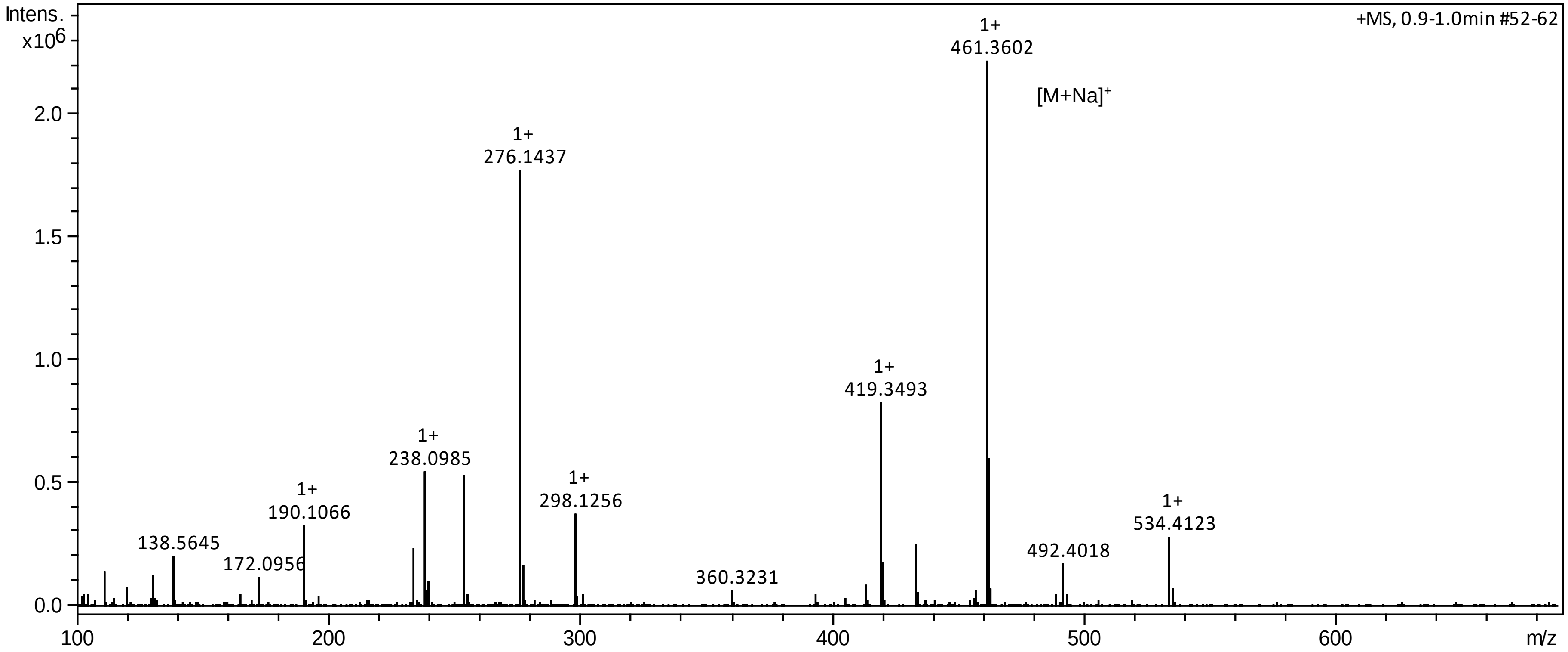

Figure S25: ^{1}H NMR spectrum (δ, $CDCl_3$, 300 MHz) of compound **3b**

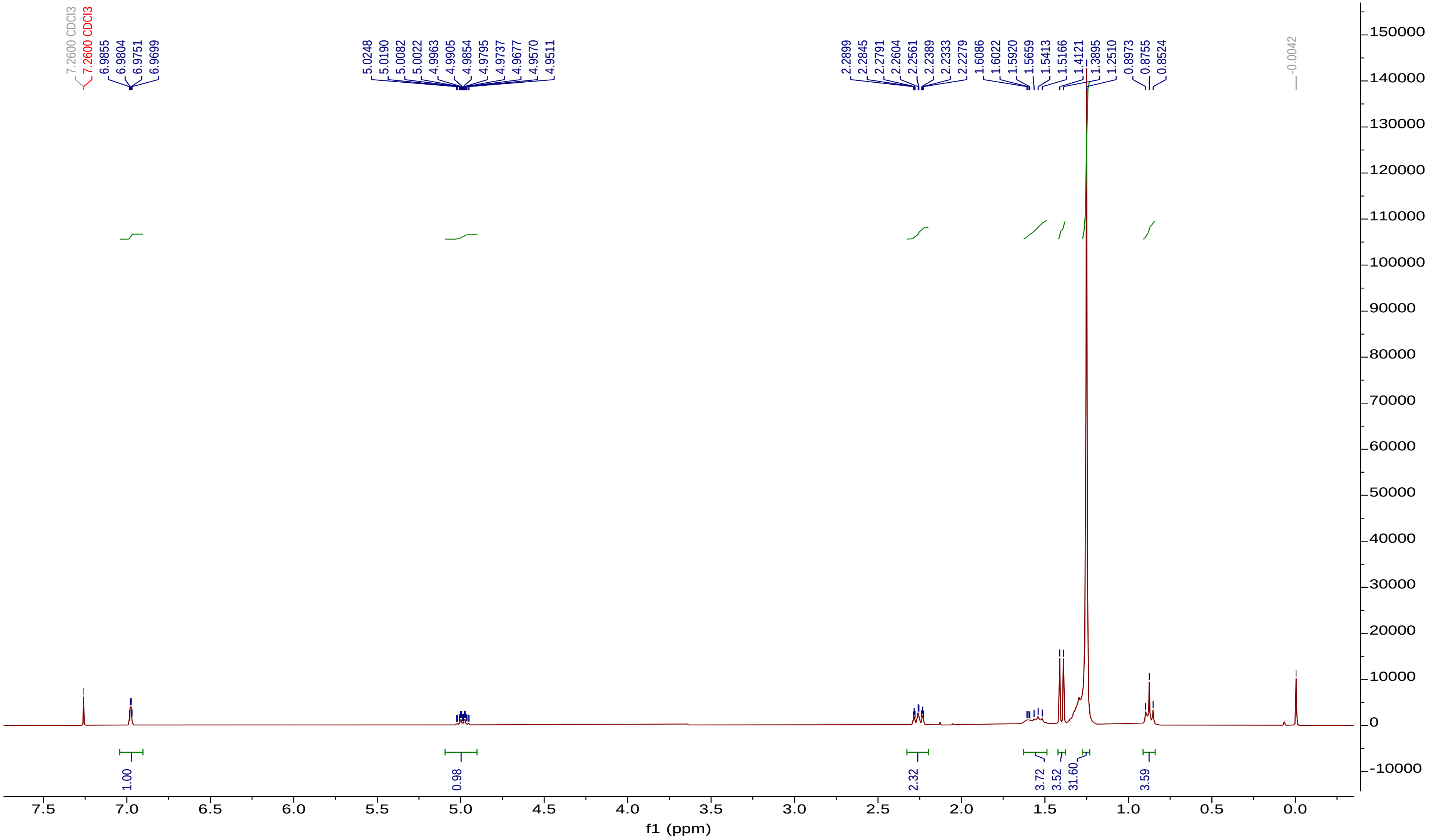

Figure S26: ^{13}C NMR spectrum (δ, $CDCl_3$, 75 MHz) of compound **3b**

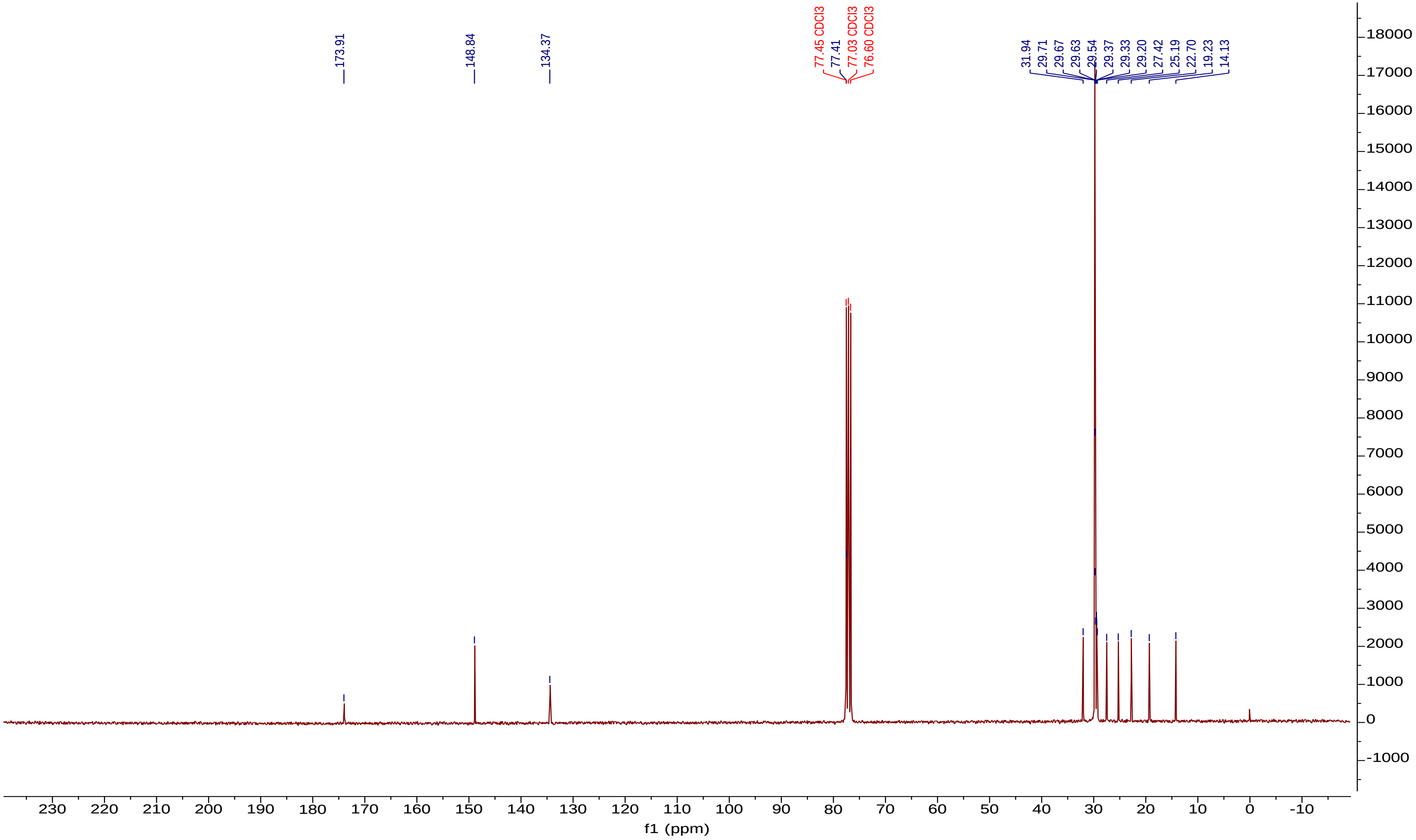

Figure S27: Mass spectrum (ESI - positive mode) - compound **3b**

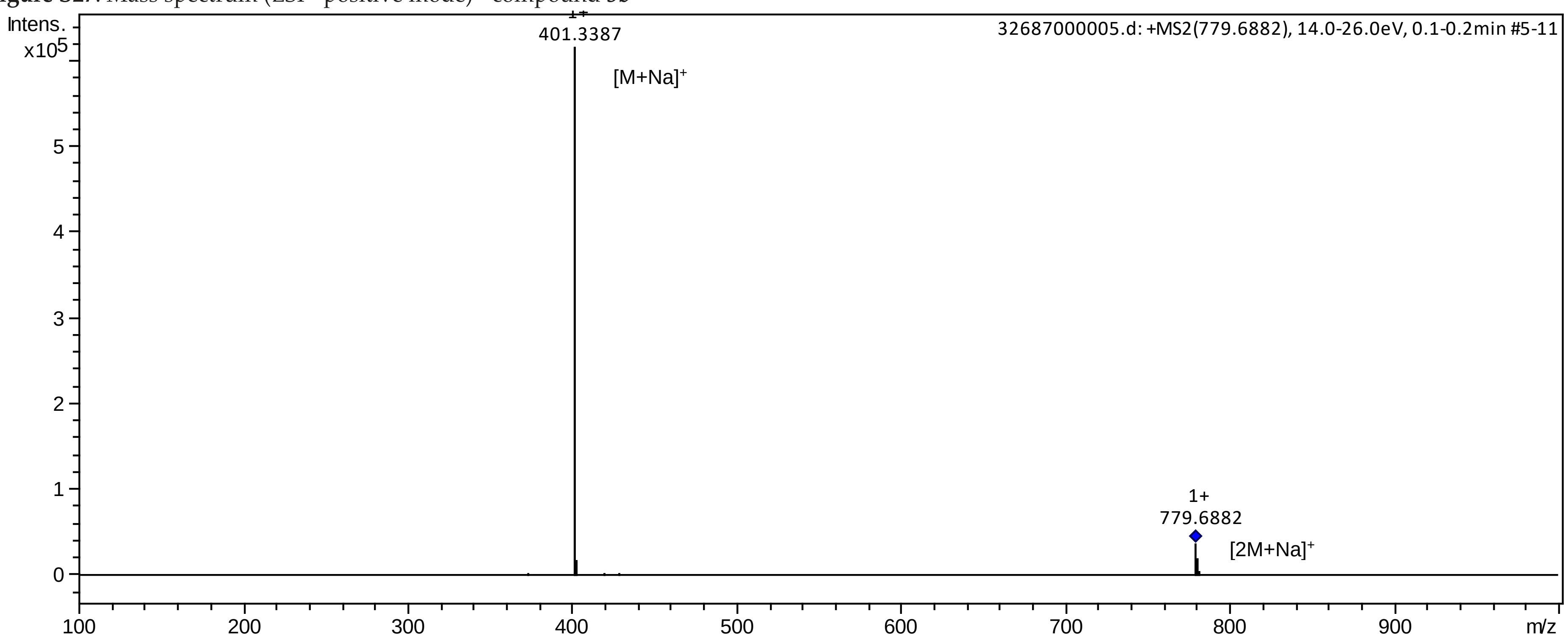

Supplement: Supplementary file 1 [file molecules-27-00893-s001.zip › molecules-1532569-supplementary.pdf]
